# Probing Antibiotic Inhibition in Small Bacterial Populations With Combinatorial Droplet Microfluidics

**DOI:** 10.1002/smsc.202500421

**Published:** 2026-01-24

**Authors:** Ashkan Samimi, Nia Verdon, Rosalind J. Allen, Miriam A. Rosenbaum

**Affiliations:** ^1^ Bio Pilot Plant Leibniz Institute for Natural Product Research and Infection Biology – Hans‐Knöll‐Institute 07745 Jena Germany; ^2^ Faculty of Biological Sciences Friedrich Schiller University 07743 Jena Germany; ^3^ Theoretical Microbial Ecology Friedrich Schiller University 07745 Jena Germany; ^4^ Cluster of Excellence Balance of the Microverse Friedrich Schiller University Jena 07745 Jena Germany

**Keywords:** antibiotic response, droplet‐based microfluidics, stochastic population dynamics

## Abstract

Bacterial infections often involve small, local populations of bacteria, yet antibiotic treatment decisions are generally based on bulk population susceptibility assays. Stochastic variability among local small populations can influence susceptibility, limiting the predictive capability of bulk assays. Therefore there is a need to better understand antibiotic response in small populations. Droplet‐based microfluidics enables the high‐throughput production of tens of thousands of picolitre droplets, in which small populations of bacteria (e.g., 8 cells) can be encapsulated and their responses to different environmental conditions tracked. Here, we use a combinatorial droplet‐generation platform, combined with microscopy and image analysis, to interrogate the responses of small populations of *Escherichia coli* to different bulk‐determined sub‐inhibitory concentrations of the antibiotics tetracycline, streptomycin, and ampicillin within a single experiment. We observe qualitatively distinct small‐population responses for these antibiotics. For the bacteriostatic ribosome‐targeting antibiotic tetracycline, growth varies nonmonotonically at low antibiotic concentrations. For the bactericidal ribosome‐targeting antibiotic streptomycin, we observe apparent bistability, some replicate populations growing while others die. For the bactericidal cell‐wall targeting antibiotic ampicillin, we observe stochastic bacterial filamentation. Our study shows how distinct phenomena impacting antibiotic susceptibility may emerge in small bacterial populations, laying a foundation for deeper studies into potential treatment implications.

## Graphical abstract

Small local populations of bacteria are often involved in bacterial infections; however, bulk susceptibility assays guide antibiotic treatment decisions. Furthermore, during the early and late stages of treatment, these small bacterial populations are exposed to sub‐inhibitory concentrations of antibiotics. Here, we utilized a combinatorial microfluidic droplet‐generation platform to evaluate the effects of bulk‐determined sub‐inhibitory antibiotic concentrations on small bacterial populations.

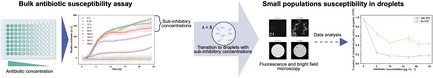

## 1 Introduction

In a clinical setting, antibiotic treatment strategies for bacterial infections are often decided based on antibiotic susceptibility assays such as disk diffusion,^[^ ^1^, ^2^, ^3^ ^]^ E‐test,^[^ ^4^ ^]^ or microbroth dilution^[^ ^5^, ^6^ ^]^ to determine the minimum inhibitory concentration (MIC).^[^ ^4^, ^7^, ^8^ ^]^ These assays test the ability of an antibiotic to inhibit “bulk” populations inoculated with tens or hundreds of thousands of bacteria.^[^ ^9^ ^]^ However, infections often involve small, local populations of bacteria, for example, within pores in the skin, cavities in bone, or inside host cells such as macrophages.^[^ ^10^ ^]^ The survival of even a small subpopulation after antibiotic treatment can reinstate the infection. Antibiotic resistance mechanisms, which are central to the current antibiotic resistance crisis,^[^ ^11^, ^12^ ^]^ can also act differently in small populations (of, for example, 8–10 bacterial cells) compared to large populations,^[^ ^13^ ^]^ and the pathways by which antibiotic resistance evolves can also be influenced by population size.^[^ ^14^ ^]^ A better understanding of how antibiotics act on small bacterial populations could, therefore, improve the effectiveness of antibiotic treatment.

Small bacterial populations may respond differently to antibiotics compared to large populations for two reasons. First, stochastic heterogeneity in the response of isogenic bacterial cells,^[^ ^15^ ^]^ e.g. due to stochastic fluctuations in gene expression,^[^ ^16^, ^17^ ^]^ becomes more important in small populations.^[^ ^18^, ^19^ ^]^ Second, due to stochastic (likely Poisson) partitioning of bacteria, considerable population size variability is expected among replicate small populations.^[^ ^13^ ^]^ These two sources of variability may combine to produce nontrivial responses at the small‐population level.

At the level of individual cells, it is well known that isogenic cells can respond differently to antibiotics. The response can even be bimodal: several studies have also shown that growth rate bistability can arise from feedback between bacterial physiology and antibiotic action.^[^ ^12^, ^20^, ^21^, ^22^, ^23^ ^]^ Phenotypic heterogeneity among individual bacteria may also have consequences for resistance evolution:^[^ ^16^ ^]^ for example, efflux pump expression is heterogeneous among cells^[^ ^24^ ^]^ and has been shown to increase the mutation rate.^[^ ^25^ ^]^ Considering population size variability, for many infections, it is well established that the infectious dose, or the number of initiating pathogenic bacteria, is a key factor determining the outcome of an infection. However, up to now the concept has mainly been studied theoretically—for example, for cooperative antibiotic resistance mechanisms^[^ ^26^ ^]^ such as beta‐lactamase production, theory suggests that local variation in bacterial density among replicate small populations can increase the effective minimal inhibitory concentration.^[^ ^13^ ^]^ Since current antibiotic susceptibility assays are not well‐suited for studying small populations, novel study technologies are required.

Droplet‐based microfluidics provides an excellent platform to investigate the response of small microbial populations to stresses. In droplet‐based microfluidics, microscale structures are used to channel the flow of two immiscible fluids (e.g., water and oil) to form nano‐to‐picoliter droplet volumes.^[^ ^27^ ^]^ The technology enables high‐throughput, monodisperse small‐volume droplet production, and its miniaturized scale allows the parallelization of experiments;^[^ ^28^, ^29^ ^]^ it has been exploited for antibiotic susceptibility assays in several studies.^[^ ^30^, ^31^, ^32^, ^33^, ^34^, ^35^, ^36^ ^]^ Bacteria can easily be encapsulated in the droplets following a Poisson loading distribution that replicates the variability of small populations during infection.^[^ ^37^ ^]^ By controlling the initial culture density, and hence the average number of bacteria per droplet, one can study the antibiotic effect on single cells^[^ ^32^, ^36^ ^]^ or on small populations.^[^ ^30^ ^]^ To investigate bacterial growth in droplets, dynamical droplet incubation methods are available; these allow droplets to remain isolated and well‐oxygenated at a desired temperature, producing bacterial growth profiles that are comparable to those of shake flasks.^[^ ^31^, ^38^ ^]^ Imaging and image analysis methods have also been developed to accurately track counts of individual bacteria within droplets over time.^[^ ^39^ ^]^ Therefore, droplet‐based microfluidic technology is a promising candidate for investigating the response of small bacterial populations to antibiotic stress.

Several previous studies have investigated the antibiotic responses of bacteria encapsulated in droplets.^[^ ^36^, ^40^ ^]^ However, these studies have focused on single‐cell droplet loading, rather than on small populations. They also have some technical limitations, lacking morphological information of bacterial cells^[^ ^36^ ^]^ or being limited to a single experimental condition per run.^[^ ^40^ ^]^ Importantly, previous studies used endpoint binary measurements for bacterial growth rather than detailed growth quantification and have focused on measuring minimal inhibitory concentration, i.e., on high antibiotic concentrations. However, to fully understand the antibiotic response, it is necessary to study not only bulk‐determined inhibitory concentrations (i.e., those above the bulk MIC) but also sub‐inhibitory concentrations (i.e., those where bulk assays show no or low inhibition of bacterial growth). At bulk‐determined sub‐inhibitory concentrations, the effect of antibiotic stress is closely coupled with bacterial growth physiology,^[^ ^21^ ^]^ potentially leading to interesting small‐population effects. Sub‐inhibitory concentrations are clinically relevant since they are encountered during the early and late phases of treatment, as antibiotic concentration at the infection site ramps up or down.^[^ ^41^, ^42^ ^]^

In this study, we investigate the response of small populations of the bacterium *Escherichia coli* to three antibiotics (tetracycline, streptomycin, and ampicillin) at a range of bulk‐determined sub‐inhibitory concentrations. These antibiotics cover several mechanisms of action—tetracycline and streptomycin are ribosome‐targeting, while ampicillin is cell‐wall targeting. All three antibiotics are, or have been, clinically relevant, and they are also well‐studied at the bulk population level (and to a lesser extent at the single‐cell level), facilitating interpretation of our results. Furthermore, all three antibiotics are technically compatible with our droplet setup, as we discuss later. We use a multiplexing droplet microfluidics platform that allows groups of droplets with different experimental conditions to be investigated simultaneously within a single experimental run,^[^ ^43^ ^]^ with droplets with specific conditions being assigned a fluorescence barcode that can be decoded via a machine‐learning approach for later analysis. In contrast to previous “linear” droplet microfluidic studies of antibiotic susceptibility, this technology provides enhanced assay reproducibility and better statistical power by using automated combinatorial sample preparation for all tested conditions and leveraging the massive scale of droplet‐based microfluidics to assess multiple conditions in a single experimental run. We combine this technology with fluorescence microscopy, allowing cell counting and morphological evaluation,^[^ ^39^ ^]^ and brightfield microscopy to assess overall growth.^[^ ^39^ ^]^ Our analysis allows us to probe how the physiological stress caused by antibiotic exposure at bulk‐determined sub‐inhibitory concentrations plays out at the small‐population level for the three antibiotics. The outcome points to the existence of novel phenomena in antibiotic response at the small‐population level and paves the way for more detailed investigations that could ultimately facilitate novel antibiotic treatment approaches.

## 2 Results and Discussion

### 2.1 Combinatorial Droplet Production for Investigating Small‐Population Responses

**Figure 1:**
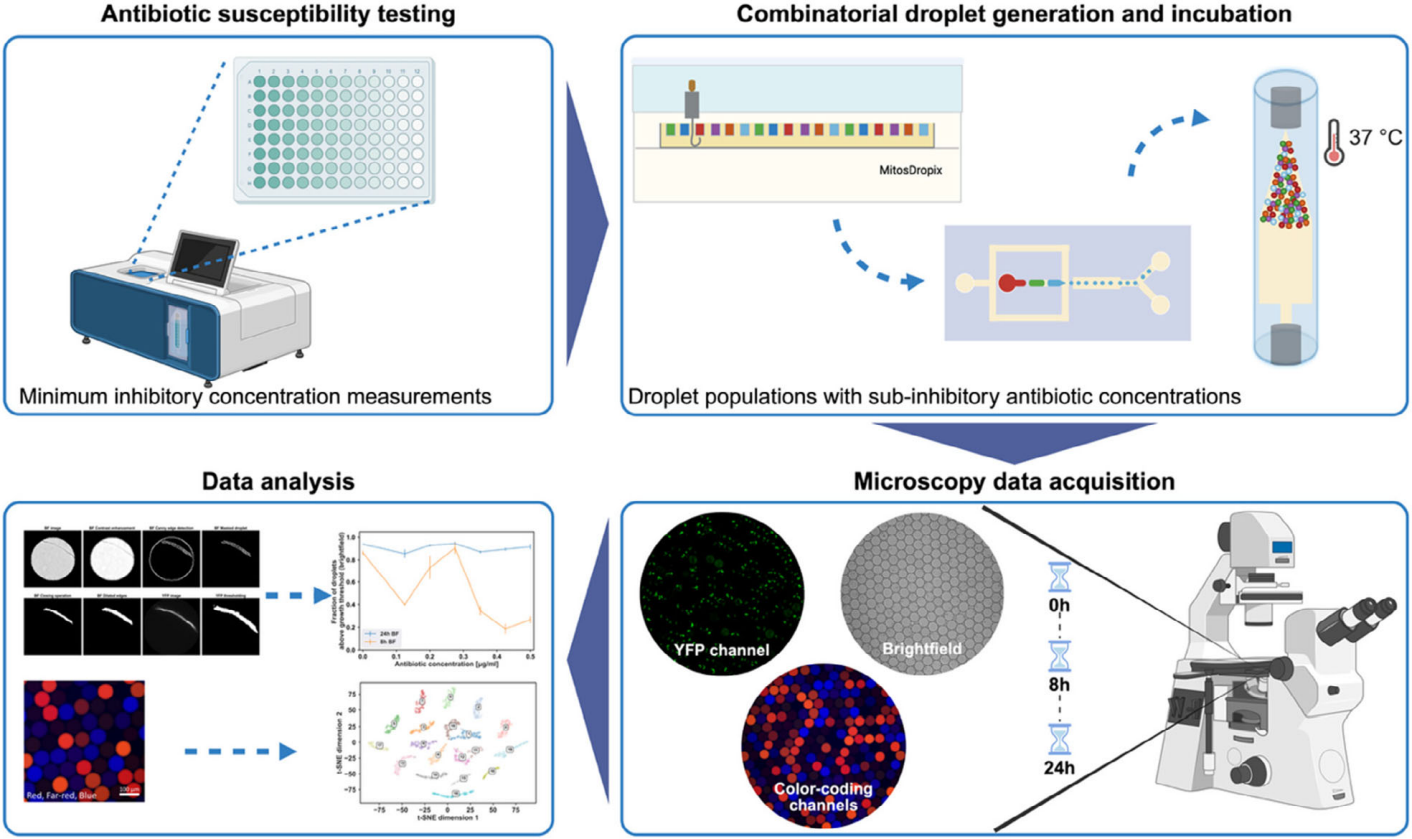
Experimental workflow for studying the stochastic response of *E. coli* small populations to antibiotics. First, a microbroth antibiotic susceptibility test in a microtiter plate is performed to find the minimum inhibitory concentrations for each antibiotic. Using the information from this step, six bulk‐determined sub‐inhibitory concentrations (i.e., no or minimal inhibition of bacterial growth) were determined for each antibiotic for the combinatorial droplet assay. Next, a droplet library of different antibiotics and concentrations is produced using the multiplexing platform (photographs of the platform are shown in Figure S2, Supporting Information).^[^ ^43^ ^]^ The droplet library is then dynamically incubated at 37 °C to achieve uniform bacterial growth conditions by providing a homogenous oxygen environment for all droplets.^[^ ^38^ ^]^ Droplets are imaged at 3 time points (0, 8, and 24 h) in the brightfield and fluorescence microscopy channels, and the data are analyzed to investigate the stochastic response of small bacterial populations to different antibiotics at varied concentrations.

**Figure 2:**
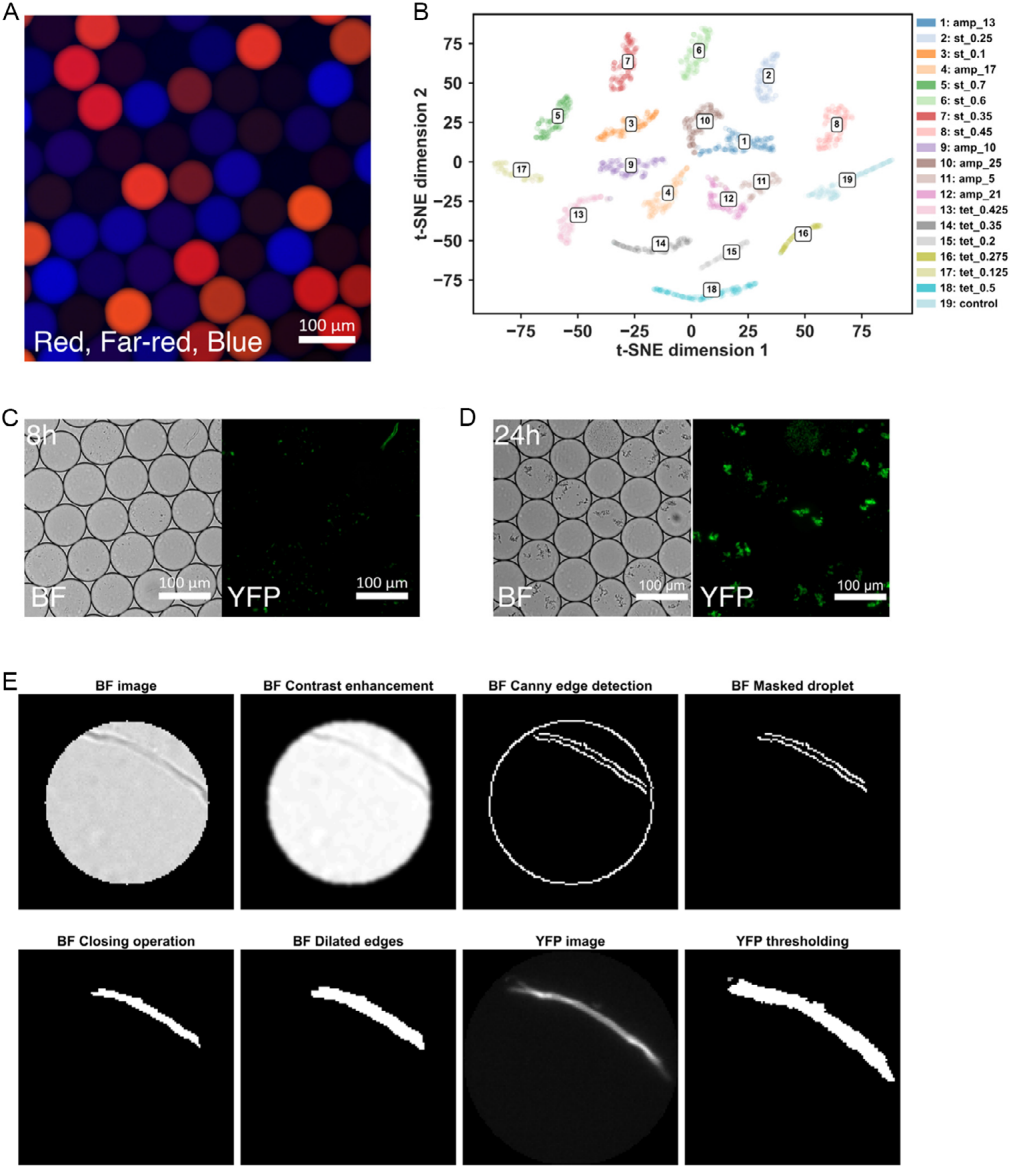
Imaging the droplet library, decoding the experimental conditions, and image analysis. Panel (A) shows a droplet library imaged in the red, far‐red, and blue fluorescence channels. The fluorescence signal in these channels encodes the experimental conditions corresponding to each droplet. (B) shows a t‐SNE plot of decoded color codes for the droplets within one experimental run imaged at 8 h. In total, 19 experimental conditions are represented in this droplet mixture; these are identified using machine‐learning algorithms,^[^ ^43^ ^]^ and the legend shows the corresponding antibiotics and concentrations corresponding to each code (Tetracycline: tet, Streptomycin: st, Ampicillin: amp). In (C) and (D), droplets imaged in the brightfield (BF) and YFP channels are shown 8 and 24 h after incubation. These images are used for analysis of bacterial growth, morphology, and killing via the image processing operations illustrated in (E) (steps for brightfield images: contrast enhancement, Canny edge detection, droplet masking, closing, and dilation; for YFP images, only intensity thresholding is applied; for full details, see the Experimental Section).

We encapsulated *Escherichia coli* bacteria carrying a constitutively‐expressed yellow fluorescence protein (YFP) marker at an average density of 8 cells per droplet (Figure S1, Supporting Information) under 18 different antibiotic conditions, incubated them under well‐aerated conditions, and used bright‐field and fluorescence microscopy to assess the response of the small populations of bacteria in the droplets to different antibiotics and concentrations. Our experiments were performed in two biological replicates. We used *E. coli* in this study because of its broad knowledge base as a model organism, its potential clinical relevance, and the availability of a fluorescent reporter strain that facilitates cell counting and the analysis of cell morphology in microscopy images. We employed a previously developed multiplexing platform (Figure S2, Supporting Information) that generates a mixture of picoliter droplets from multiple samples representing different experimental conditions in a single experimental run (here, different antibiotics and concentrations).^[^ ^43^ ^]^ In this platform, fluorescent dyes are utilized to color‐code the different experimental conditions within the droplet library produced by the multiplexing platform.^[^ ^43^ ^]^ One can choose up to 91 color codes produced by combining red, far‐red, and blue.^[^ ^43^ ^]^ In our experimental design, we used red and far‐red dyes, and a defined concentration of either of these dyes was added to the cultivation medium, the bacterial cell solution, and the stock concentrations of antibiotics. For the concentration used for each antibiotic and dye, see the Experimental Section. For each experimental condition, a 10 μL sample was prepared for droplet generation by combining cultivation medium, stock antibiotic concentration, and bacterial inoculum. As a result, each solution has a unique color code, representing the applied antibiotic type and concentration (**Figure** **1** and **2**B). Here, 19 experimental conditions were simultaneously tested, which included 3 antibiotics, tetracycline, streptomycin, and ampicillin, each at 6 different bulk‐determined sub‐inhibitory concentrations (i.e., no or low inhibition of bacterial growth) plus one control condition without any antibiotics. The droplet library was dynamically incubated at 37 °C to ensure optimal aeration.^[^ ^38^ ^]^ Droplets were imaged in fluorescence and brightfield channels after 0, 8, and 24 h of incubation (Figure 1).

### 2.2 Data Analysis Pipeline for the Identification of Experimental Conditions and Quantifying the Behavior of Bacterial Cells

Droplets corresponding to different experimental conditions were identified within the droplet library using images taken in the red, far‐red, and blue fluorescence channels (Figure 2A) and employing a machine‐learning approach.^[^ ^43^ ^]^ The identified droplet populations are visualized in Figure 2B.

The brightfield and YFP microscopy images were used to measure bacterial growth/killing. These two contrasting approaches were used to quantify different aspects of bacterial growth within a single droplet. From the brightfield images, we quantified overall bacterial density within individual droplets (Figure 2C,D) via a series of image processing operations, resulting in a final binary image in which the area covered by bacteria was used as a measure of growth (Figure 2E). In parallel, the YFP fluorescence images were used to count the number of metabolically active bacterial cells, assign a metabolically active/not active label, and evaluate changes in bacterial morphology (Figure 2C,D) in each droplet. These images were processed by thresholding to remove background noise, followed by steps to identify and count objects corresponding to cells (Figure 2E). Further details on the image processing steps are given in the Experimental Section, while Table S1 to S3, Supporting Information, show the number of droplets analyzed. It should be noted that the information from the two quantification approaches is complementary, as they quantify intrinsically different properties of the bacterial growth within each droplet. For example, the YFP image analysis allows us to complement and interrogate the findings of our brightfield analysis by quantifying the number of metabolically active bacteria and the presence of filamentous cells, which are not accessible in the brightfield analysis. Nevertheless, it is of course expected that the two methodologies correlate with each other. Therefore, we correlated the brightfield and YFP outputs across all experimental conditions, for both biological replicates (Figure S3–S14, Supporting Information). As expected, we observe a positive correlation between the two analysis approaches, with some variability since different aspects of bacterial behavior are measured by the two approaches. The variability may also originate from bacterial cell aggregation and antibiotic‐induced changes, such as bacterial cell lysis, which manifest differently in bright‐field vs YFP images.

### 2.3 Nonmonotonic Dependence of Growth on Antibiotic Concentration for a Small Bacterial Population Exposed to Bulk‐Determined Sub‐Inhibitory Tetracycline

**Figure 3:**
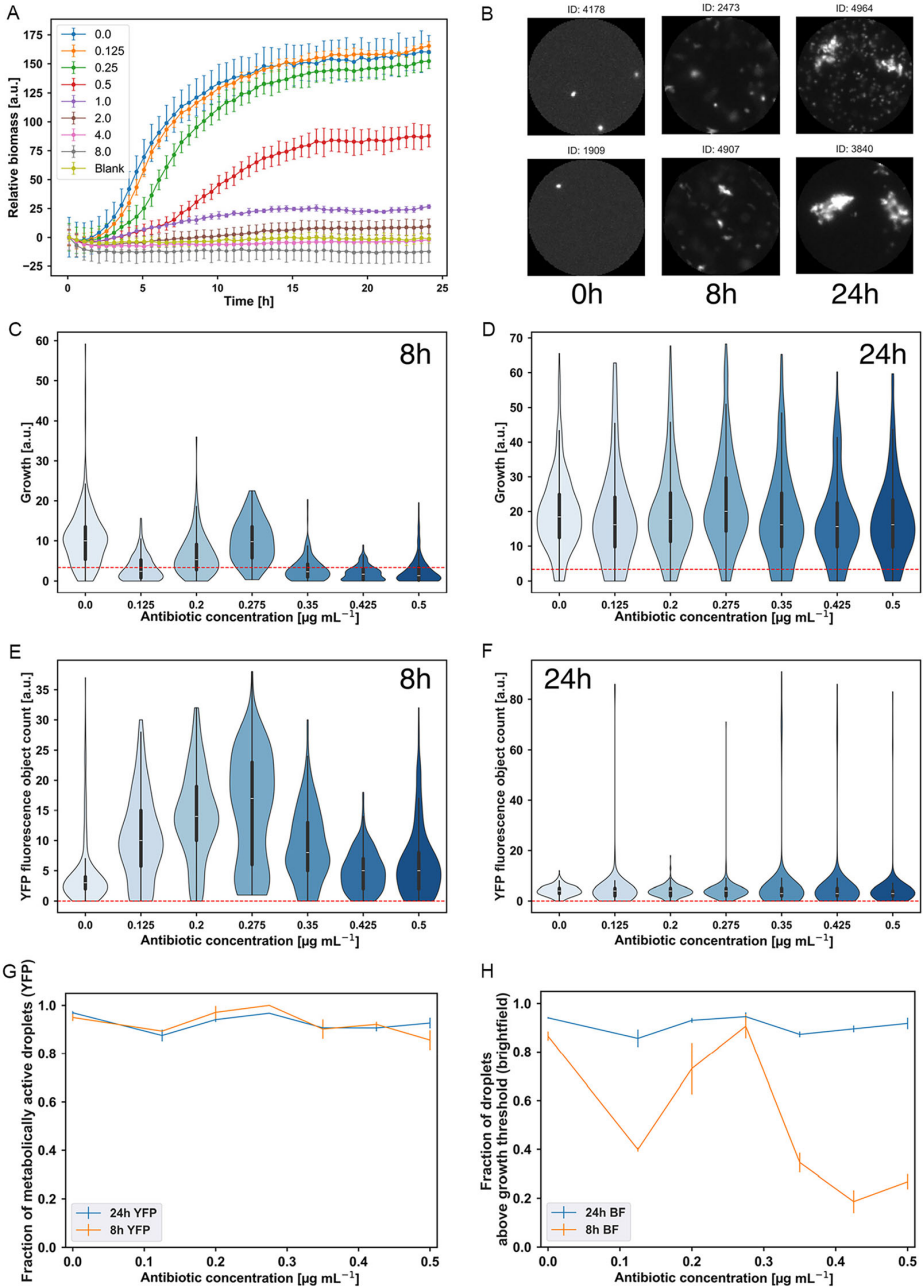
Tetracycline susceptibility of our droplet populations (results are shown for biological replicate 1). Panel (A) shows the results of the microtiter plate bulk culture susceptibility assay. The minimum inhibitory concentration of tetracycline based on this assay for our *E. coli* strain is 4 μg mL^−1^. (B) shows randomly selected droplet images at 0.275 μg mL^−1^ for three time points to give an overall impression of the data. In (C) and (D), violin plots for the growth of individual droplets exposed to different tetracycline concentrations are shown after 8 and 24 h of incubation. The dashed red line is the threshold calculated to identify droplets with an increased bacterial biomass (=3.4 for biological replicate 1). (E) and (F) show YFP cell counts after 8 and 24 h of incubation; the dashed red line shows the threshold for metabolic activity (a count above zero); see panel (G), which shows the fraction of metabolically active droplets. (H) shows the fraction of droplets exhibiting higher growth than the threshold from brightfield image analysis (dashed red line in (C) and (D)). Error bars were calculated by randomly splitting the dataset for each antibiotic concentration into three sub‐sets and reporting the standard deviation in the mean across the three sub‐sets. Minimum droplet number *n* = 127 and maximum *n* = 439 (see Table S1, Supporting Information).

Tetracycline is a bacteriostatic ribosome‐targeting antibiotic that targets the ribosomal 30S complex, inhibiting the binding of aminoacyl tRNA and slowing growth.^[^ ^21^, ^44^ ^]^ To establish a suitable concentration range for investigating the response of small bacterial populations to sub‐inhibitory tetracycline, we initially performed a bulk microtiter plate cultivation with twofold antibiotic dilutions. This showed that, in bulk, the growth of our strain is entirely eliminated at 4 μg mL^−1^ tetracycline, with significant inhibition already at 0.5 μg mL^−1^ (**Figure** **3**A). To investigate sub‐inhibitory growth dynamics in small populations, we therefore used tetracycline concentrations in the range 0–0.5 μg mL^−1^ for the multiplexed droplet experiment. We used loading conditions for which we expect very few droplets to be empty at the start (see Experimental Section). Figure S1, Supporting Information, shows the expected occupancy (cell count within droplets) for both biological replicates, calculated from the known inoculum density using a Poisson distribution. Since we cannot count cells in droplets immediately after loading in this setup, we verify the high level of droplet occupancy by checking that the control population shows growth in all droplets after 8 h (Figure 3G,H).

We quantified overall growth in each droplet using our brightfield microscopy images, defining growth as the area covered by bacterial cells within a droplet image (see Experimental Section). To investigate the effect of bulk‐determined sub‐inhibitory tetracycline on small *E. coli* populations in more detail, we plotted the distribution of growth values among droplets exposed to a given tetracycline concentration (Figure 3C,D). After 8 h, we observed suppression of growth compared to the uninhibited control for most of the tetracycline concentrations (Figure 3C). Strikingly, however, the dependence of growth on tetracycline concentration was nonmonotonic—at the intermediate concentration of 0.275 μg mL^−1^ tetracycline, growth is similar to the uninhibited control. This unexpected behavior was reproduced in an independent biological replicate experiment (Figure S20, Supporting Information). The effects of tetracycline on growth are transient, since after 24 h of incubation, the distribution of growth values is similar to the control across all antibiotic concentrations (Figure 3D), consistent with the expected bacteriostatic action of tetracycline, i.e., slowing growth but not killing.

Analysis of droplet images in the YFP channel revealed normal cell morphology on exposure to tetracycline (Figure 3B shows images at 0.275 μg mL^−1^; see also Figure S15–17, Supporting Information, for randomly chosen images of control droplets and other tetracycline concentrations). Automated morphological analysis (see Methods) did not reveal any filamentation, although previous reports suggest that tetracycline can cause filamentation in some *E. coli* strains (Figure S18, Supporting Information).^[^ ^45^ ^]^ The fact that cell morphology remains normal is consistent with the expected mode of action of tetracycline, which is inhibition of protein synthesis,^[^ ^46^ ^]^ although effects on the cell wall and DNA synthesis have been reported at higher concentrations.^[^ ^47^, ^48^ ^]^ Analysis of our YFP images also suggested that tetracycline‐treated populations showed a reduced tendency to aggregate compared to the uninhibited control populations (Figure S19, Supporting Information). To quantify this effect better and to further understand the effects of tetracycline on small populations, we obtained counts of the number of fluorescent objects in each droplet from our YFP images. These counts are expected to reflect the number of metabolically active cells since metabolically inactive cells are not expected to fluoresce. However, the presence of bacterial aggregates can lead to a low count even when there is high growth. This is the case in the uninhibited control, where aggregation is extensive (Figure S15, Supporting Information)—here, fluorescent object counts are low (Figure 3E), but growth is high (Figure 3C). The observed reduction in aggregation upon antibiotic exposure might be associated with an antibiotic‐induced enhancement of cell motility.^[^ ^49^ ^]^ Despite this confounding factor for the uninhibited control, our YFP counts confirm the observation from our brightfield images that the transient (8 h) effects of tetracycline are nonmonotonic with concentration (Figure 3E and S20, Supporting Information), but after 24 h of incubation, counts are similar across all concentrations (Figure 3F and S20, Supporting Information).

Tetracycline is a bacteriostatic antibiotic that is expected to slow growth but not to kill cells. As a proxy for the presence of metabolically active cells, we used our YFP analysis to quantify the fraction of droplets showing any (1 or more) fluorescent objects. The fraction of droplets showing metabolic activity after antibiotic exposure reflects survival in the presence of the antibiotic. This analysis revealed that, as expected, almost all droplets remain metabolically active across the whole range of tetracycline concentrations (Figure 3G). We also performed a parallel analysis with our brightfield images, in which we defined a threshold growth value (red dashed line in the violin plots of Figure 3C,D, based on the average growth value for brightfield images at the zero time point) to separate droplets where no growth was detected from those exhibiting growth. We could then determine the fraction of droplets containing detectable bacterial biomass based on the brightfield image analysis data. As shown in Figure 3H, after 8 h incubation, this fraction varies nonmonotonically with antibiotic concentration, while after 24 h incubation, the nonmonotonic response is lost (Figure 3H).

The qualitative difference between the fraction of metabolically active droplets (Figure 3G) and the fraction of growing droplets (Figure 3H) arises from the different thresholds used in these analyses. A droplet is defined as metabolically active if it contains a single detected YFP object, but to be defined as growing, it needs to show biomass above a nonzero threshold. Therefore, the slowing of growth by tetracycline alters the fraction of “growing” droplets in the brightfield analysis but does not affect the fraction of “metabolically active” droplets in the YFP analysis. Taken together, the two analyses indicate that tetracycline at these concentrations does not kill, but it slows growth during the initial 8‐hour time window in a manner that is non‐monotonic in the antibiotic concentration.

The striking nonmonotonicity of the growth response in droplets, where tetracycline slows growth for most concentrations, but the inhibition is relieved at 0.275 μg mL^−1^ in both biological replicates (Figure 3C,G, and S20, Supporting Information), appears to be an example of a poorly understood phenomenon known as hormesis, in which low doses of toxic substances such as antibiotics can stimulate growth.^[^ ^50^, ^51^ ^]^ Hormesis has previously been reported for *E. coli* strain MG1655 exposed to low‐dose tetracycline in bulk cultures, for concentrations around 0.015 μg mL^−1^,^[^ ^51^ ^]^ much lower than our lowest bulk concentration. Therefore, we speculate that this phenomenon might be shifted to a much higher concentration range for small populations in the confined droplet environment. The mechanisms underlying hormesis are unclear.^[^ ^50^, ^51^ ^]^ One possible mechanism could be the activation of efflux pumps that export tetracycline from the bacterial cell;^[^ ^41^, ^52^ ^]^ sub‐inhibitory antibiotic concentrations (here 0.275 μg mL^−1^) may upregulate the expression of efflux pumps,^[^ ^53^ ^]^ while at higher concentrations, the efflux might be insufficient to protect from the antibiotic. However, further investigation will be needed to determine the mechanism underlying hormesis in our experiments and to understand how this phenomenon is affected by small‐population size.

### 2.4 Streptomycin Produces a Bimodal Response in Small Bacterial Populations

**Figure 4:**
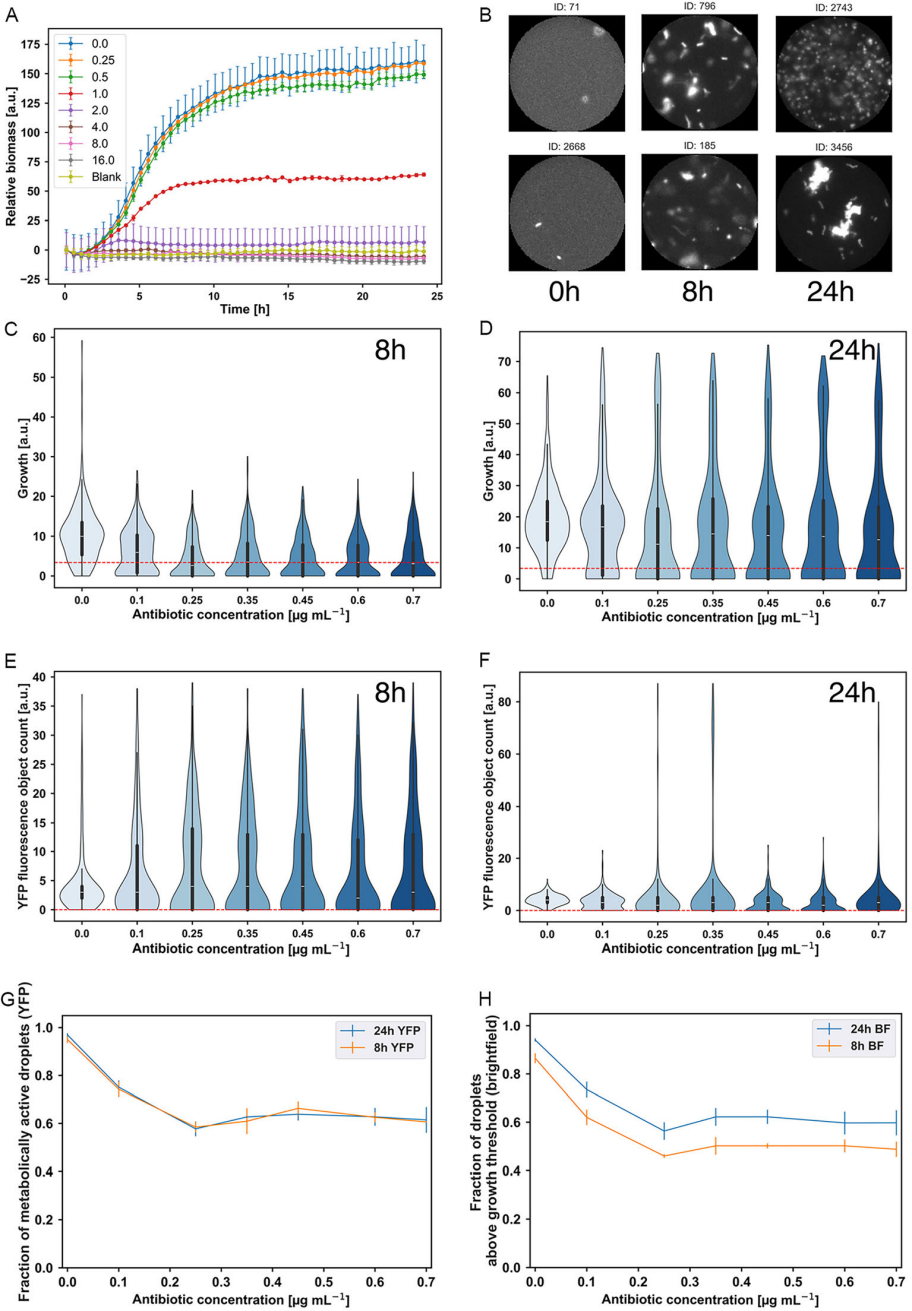
Streptomycin susceptibility of our droplet populations (results are shown for biological replicate 1). Panel (A) shows the results of bulk culture microtiter plate assays for a range of antibiotic concentrations. The minimum inhibitory concentration is 4 μg mL^−1^. (B) shows images of randomly selected droplets in the YFP channel at 0.35 μg mL^−1^ for the three different incubation time points. Violin plots for the growth of individual droplet populations after 8 and 24 h of incubation, based on brightfield image analysis, are shown in (C) and (D). The dashed red line indicates the threshold used to distinguish empty and filled droplets, as explained in the main text (the threshold is 3.4). Violin plots for counts of metabolically active bacteria, obtained from the YFP images, are shown in (E) and (F) for 8 and 24 h of incubation. The red dashed line shows the threshold for defining metabolic activity (1 or more active bacteria). The fraction of droplets showing metabolic activity (from the YFP analysis) is presented as a function of streptomycin concentration in (G). The fraction of droplets exhibiting growth higher than the brightfield threshold is shown in (H). Error bars were calculated by randomly splitting the dataset for each antibiotic concentration into three sub‐sets and reporting the standard deviation in the mean across the three sub‐sets. Minimum droplet number *n* = 229 and maximum *n* = 437 (see Table S2, Supporting Information).

Streptomycin belongs to the bactericidal aminoglycoside group of ribosome‐targeting antibiotics. Streptomycin interrupts the ribosome cycle at the initiation of protein synthesis, eventually causing cell death, although the actual killing mechanism remains somewhat unclear.^[^ ^54^ ^]^ Our bulk microtiter plate antibiotic susceptibility assays using a twofold dilution series of streptomycin revealed that the minimum inhibitory concentration of streptomycin for our *E. coli* strain was 4 μg mL^−1^ (**Figure** **4**A), with a significant reduction in biomass already at 1 μg mL^−1^. Therefore, to investigate sub‐inhibitory effects of this antibiotic on small populations, we chose a concentration range of 0.1–0.7 μg mL^−1^ for our multiplexed droplet experiments.

Automated morphological analysis of our YFP images showed mostly typical cell morphologies, suggesting that streptomycin does not cause significant filamentation in our *E. coli* strain, consistent with the antibiotic targeting protein synthesis (Figure S21, Supporting Information; see the Experimental Section for a detailed explanation). Figure 4B shows sample images of droplets exposed to 0.35 μg mL^−1^ streptomycin; more images of randomly chosen droplets at different concentrations are shown in Figure S22 and S23, Supporting Information.

The overall growth of bacterial cells exposed to (bulk‐determined) sub‐inhibitory streptomycin was quantified using our brightfield microscopy images. Violin plots for growth at different streptomycin concentrations after 8 h of incubation indicate the existence of two droplet sub‐classes—droplets that are empty and those that exhibit growth; the latter droplets show a gradual reduction in the growth value with increasing antibiotic concentration (Figure 4C). After 24 h of incubation with antibiotic, we observe the appearance of a third subclass of droplets with apparently higher growth values than the control (Figure 4D). Examination of droplet images shows that this phenomenon is due to cell dispersal: in the droplets with apparently very high growth values, cells have dispersed, whereas the control droplets show large aggregates (Figure 4B). This effect may be consistent with previous reports that sub‐inhibitory concentrations of streptomycin can enhance bacterial motility.^[^ ^55^, ^56^ ^]^ Overall, our results indicate a bimodal response to streptomycin: a small droplet‐encapsulated population of bacteria either continues to grow in the presence of the antibiotic or is completely inhibited. The appearance of droplets showing complete inhibition is consistent with the expectation that streptomycin is bactericidal, i.e., it causes bacterial death; however, the fact that some droplet populations are killed at these antibiotic concentrations while others continue to grow is surprising.

To further understand the effects of (bulk‐determined) subinhibitory concentrations of streptomycin, we also analyzed our YFP images. Consistent with our brightfield observations (Figure 4D), our YFP images showed a bimodal response to the antibiotic, evidenced by the appearance of distinct subclasses of droplets with or without metabolically active cells (Figure 4E). Also consistent with our brightfield results, fewer bacterial aggregates were observed in streptomycin‐treated droplets compared to the antibiotic‐free control (Figure S24, Supporting Information). This is also apparent in the appearance of a droplet subpopulation with very high fluorescent cell counts in Figure 4E, corresponding to dispersed cells (however, this subpopulation is diminished after 24 h of exposure to streptomycin, suggesting that cells aggregate at late times (Figure 4F)).

To investigate the bimodal response to streptomycin, we quantified the fraction of droplets that showed either growth (from our brightfield images) or metabolic activity (from our YFP images) using the previously discussed thresholds. Our results were broadly consistent across the different quantification methods and for either 8 h or 24 h of antibiotic exposure (Figure 4G,H): for low streptomycin concentrations (approximately below 0.2 μg mL^−1^), the fraction of growing/active droplets decreased with increasing antibiotic concentration. However, for higher concentrations, ≈50%–60% of droplets show growth/activity independent of the antibiotic concentration. Two significant conclusions can be drawn from these results. First, sub‐inhibitory streptomycin causes bacterial killing, since growth/metabolic activity is extinguished in a significant fraction of droplets. Second, the killing appears to be stochastic since around half of the droplet populations are not killed, even at the higher antibiotic concentrations, and indeed show growth approximately comparable to the antibiotic‐free control. Similar results were obtained for an independent biological replicate experiment (Figure S25, Supporting Information).

Taken together, our results show that bulk‐determined sub‐inhibitory concentrations of streptomycin reduce average bacterial cell viability (Figure 4G and S23, Supporting Information), but this is a stochastic effect whereby replicate small populations can respond very differently, with some being hardly affected by the antibiotic while others are killed. Notably, as shown in Figure 4A, this variability in outcomes could not be deduced from bulk susceptibility assays. For those droplet populations that continue to grow, we also observe variability in bacterial aggregation, with some droplets containing dispersed cells while others contain aggregates (Figure 4C–F, and S25C,D, Supporting Information).

Interestingly, a bistable response of individual cells to streptomycin has been predicted theoretically in a model in which antibiotic influx and ribosome binding are irreversible, such that the fate of the cell is determined by the relative rates of antibiotic‐ribosome binding versus new ribosome synthesis.^[^ ^21^ ^]^ This bistable response has not yet been verified experimentally, but could be consistent with our observations that some small populations are killed while others are not. In the future, similar experiments with single‐cell inoculation could be used to link individual cell responses to those of small populations and hence understand quantitatively the stochasticity of killing versus survival.

### 2.5 Changes in Bacterial Morphology When Small Populations are Exposed to Bulk‐Determined Sub‐Inhibitory Concentrations of Ampicillin

**Figure 5:**
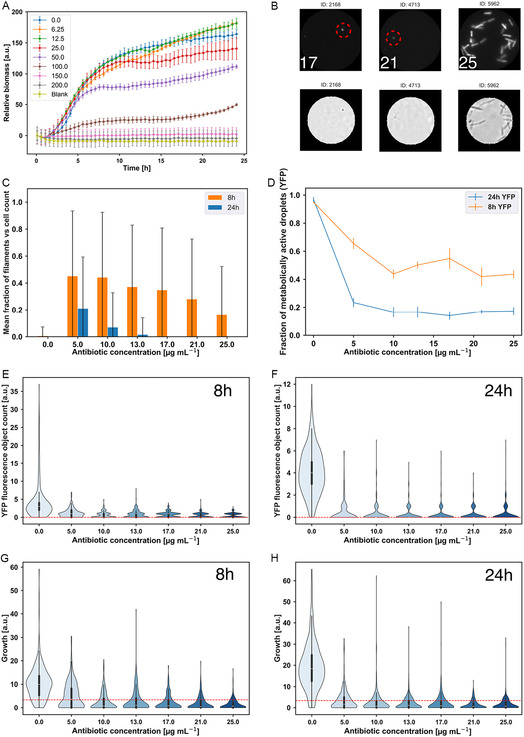
Ampicillin susceptibility of our droplet populations (results are shown for biological replicate 1). Panel (A) shows bulk cultivation microtiter plate experiments for a range of ampicillin concentrations. Based on these results, the minimum inhibitory concentration for this antibiotic is 150 μg mL^−1^. (B) shows metabolically active cells in droplets after 24 h for three high concentrations of ampicillin in YFP and brightfield channels (replicate 1; the numbers indicate antibiotic concentration in μg mL^−1^). The single, small cells (indicated by red circles) might be persisters. (C) shows the fraction of cells within a droplet that are filamentous; this fraction is averaged over all droplets. The error bars indicate the standard deviation across droplets, cut at zero. (D) shows the fraction of droplets that are defined as metabolically active based on the YFP image data (i.e., that contain at least one YFP object). Error bars indicate the standard deviation. (E) and (F) show violin plots of the YFP cell counts of droplets after 8 and 24 h of incubation. (G) and (H) show violin plots of the growth after 8 and 24 h of incubation, based on brightfield image analysis. The dashed red line indicates the threshold for identifying growth exhibiting droplets (=3.4 for biological replicate 1 brightfield images). Minimum droplet number *n* = 177 and maximum *n* = 422 (see Table S3, Supporting Information).

Ampicillin belongs to the beta‐lactam class of antibiotics, which target penicillin‐binding proteins (PBPs) involved in the synthesis of the peptidoglycan cell wall during elongation and division.^[^ ^57^, ^58^, ^59^ ^]^ These antibiotics often cause morphological changes: inhibition of elongation leads to cell rounding, while inhibition of division leads to the formation of long, filamentous cells. Exposure to ampicillin is often observed to cause filamentation.^[^ ^58^ ^]^ In Gram‐negative bacteria such as *E. coli,* beta‐lactam antibiotics cause cell killing via lysis.^[^ ^60^ ^]^ Bulk susceptibility assays for our *E. coli* strain exposed to ampicillin demonstrated a relatively high minimal inhibitory concentration of 150 μg mL^−1^ with a reduction in bacterial biomass from 50 μg mL^−1^ (**Figure** **5**A). To investigate the bulk‐determined sub‐inhibitory effects of ampicillin in small populations, we chose a concentration range of 5–25 μg mL^−1^.

As expected, analysis of YFP images of our droplet populations exposed to ampicillin showed changes in bacterial morphology: most growth‐exhibiting droplets contained filaments (Figure 5B right images and Figure 5C; see also Figure S26 and 27, Supporting Information). Quantifying the fraction of cells within a given droplet that are filaments (defined as the number of counted cells with a size greater than 200 pixels, divided by the total counted cells; see Experimental Section), we observed that the mean proportion of filamentous cells (averaged across droplets) was high after 8 h exposure to ampicillin (≈45% of cells at 5 and 10 μg mL^−1^), but after 24 h of incubation, it became much lower (Figure 5C). At this time point, we also see significant bacterial lysis leading to cell death and the absence of YFP production. This suggests a picture in which cells initially filament in response to the antibiotic, but the filaments (as well as nonfilamentous cells) eventually lyse after prolonged exposure. Indeed, bacterial lysis is clearly apparent in Figure S28, Supporting Information, where selected droplets show no YFP intensity—implying no metabolic activity—while cell biomass remnants are still visible in the brightfield images of the same droplets. We note that filamentous cells are also occasionally observed in the control droplets, but at a much lower frequency than for droplets exposed to ampicillin (Figure 5C and S29G, Supporting Information).

In our droplet populations, most of the droplets showed inhibition at much lower concentrations of ampicillin than in our bulk susceptibility assays. Analysis of our YFP images showed that for ampicillin concentrations above 5 μg mL^−1^, metabolic activity was lost in around half of the droplets after 8 h incubation, and in more than 80% of droplets after 24 h of incubation (Figure 5D). The YFP cell count distributions also support this picture (Figure 5E–F). For all ampicillin concentrations tested, the droplet populations are typically reduced to just a few metabolically active (fluorescent) cells after 8 h of incubation, and after 24 h, we observe only a few droplets with more than one or two fluorescent cells. Loss of metabolic activity in most droplets is consistent with the expected bactericidal effect of ampicillin; however, the fact that a minority of our small droplet populations maintain metabolic activity is intriguing. Focusing on those droplets that retained a signal in the YFP channel at higher concentrations of ampicillin, we observed the presence of single, very small cells (Figure 5B left and middle images). We speculate that these might be persister cells—i.e., a subpopulation of antibiotic‐tolerant, potentially slow‐growing physiological variants.^[^ ^61^, ^62^ ^]^ Interestingly, we also observed a few droplets that contained larger numbers of metabolically active, apparently growing cells, even at the highest ampicillin concentration tested (Figure S29; and 5B, Supporting Information, right image). These might be antibiotic‐resistant cells that are able to proliferate in the presence of ampicillin, although our present setup does not allow for extraction and further testing of these droplets. It is conceivable that the presence of a subpopulation of such resistant cells might explain the very different concentration range needed to inhibit our bulk cultures compared to our droplet populations. Therefore, our experiments reveal heterogeneous, stochastic responses to ampicillin among small droplet populations that could arise from pre‐existing physiological heterogeneity within the bacterial population. This leads to (on average) a much higher susceptibility for bacteria in small populations compared to the bulk.

To further investigate the effects of ampicillin on our droplet populations, we also quantified growth based on the brightfield images. Consistent with our YFP analysis, growth drastically decreased with increasing antibiotic concentration (Figure 5G,H). Quantification of the fraction of droplets exhibiting growth higher than the brightfield threshold confirms this picture: after either 8 h or 24 h of incubation, around 90% of control droplets show growth, whereas for the lowest applied concentration of ampicillin (5 μg mL^−1^) only 50% of the droplet are growing and for the higher antibiotic concentrations this decreases to around 20% (Figure S30, Supporting Information).

Taken together, our results show that ampicillin causes cells in droplets to filament, consistent with previous reports^[^ ^63^ ^]^ and its known mode of action; filamentation may delay cell lysis but does not prevent it. More surprisingly, the population‐level effects of ampicillin are very different in droplets compared to bulk experiments. Ampicillin eliminates metabolic activity from a significant fraction of the small droplet populations even at the lowest concentration tested (5 μg mL^−1^) after 24 h of incubation, whereas growth continues in our bulk assays up to at least 50 μg mL^−1^. We do not expect nutrient or oxygen supply to be different between our bulk and droplet cultivations.^[^ ^38^ ^]^ We also would not a priori expect inoculum effects^[^ ^64^ ^]^ to differ between droplets and bulk since the starting cell density for both the microtiter plate well and droplet assays was 40 × 10^6^ CFU mL^−1^, which resulted in 8 × 10^6^ cells per 200 μL in the well and an average of about 8 cells per droplet (Figure S1, Supporting Information). Recent work shows that the collective behavior of microbes in small populations can be different compared to large populations—in particular, antibiotic‐degrading ß‐lactamase enzymes^[^ ^65^ ^]^ can provide more effective protection in fragmented habitats.^[^ ^13^ ^]^ However, our strain is not expected to produce ß‐lactamase, and our results suggest the opposite phenomenon—the fragmented droplet population is apparently more susceptible to ampicillin. Other collective behaviors, such as cell‐to‐cell signaling leading to the induction of resistance mechanisms,^[^ ^66^ ^]^ could perhaps provide protection for the bulk population but not the droplet population. Alternatively, an explanation might lie in cell‐to‐cell heterogeneity within the bacterial population. Our droplet experiments suggest that the population contains a small number of pre‐existing resistant cells (Figure S29, Supporting Information). In the presence of the antibiotic, these cells might outgrow the majority of susceptible cells in a bulk experiment, whereas in a droplet experiment, they remain confined within individual droplets. Further work, for instance, with single‐cell inoculation, will be needed to determine in detail how ampicillin acts differently on bulk versus fragmented populations.

## 3 Conclusion and General Discussion

In this study, we demonstrated how the combination of multiplexed droplet‐based microfluidics with brightfield and fluorescence microscopy, image processing, and machine‐learning algorithms can be used to investigate the effects of sub‐inhibitory antibiotic exposure (as determined from bulk assays) on small bacterial populations. The use of antibiotic concentration ranges defined as subinhibitory in bulk assasy is a first step in investigating small‐population responses; in future studies, one could refine the concentration range based on our droplet‐based data. Our results reveal that these antibiotic concentrations indeed have different effects on small populations compared to bulk populations and that the nature of these differences is antibiotic‐dependent. For tetracycline, we observed hormesis, i.e., the stimulation of growth by the antibiotic at low concentrations, apparently in a different concentration range compared to reports of this effect in bulk populations. This result might be clinically relevant in situations where antibiotic concentrations are very low, such as environmental pollution of drinking water (or, in animals, with the use of low‐level antibiotics as a growth stimulant). For streptomycin, we observed bimodality, with some droplet populations being killed while others continued to grow, even at the highest antibiotic concentrations tested. This could have significant implications for the interpretation of the minimal inhibitory concentration in fragmented versus bulk populations, which should be investigated in further work. Both tetracycline and streptomycin also reduced the tendency for bacteria to aggregate in the droplets, compared to the antibiotic‐free control. Ampicillin caused filamentation as expected and reduced the viability of small populations considerably more than that of bulk populations, possibly due to differences in how pre‐existing heterogeneity in resistance plays out in fragmented versus bulk populations. This finding also has potential clinical implications, since it might suggest that susceptibility measurements made in bulk may not apply in real infections where a population is fragmented.

Our study focused on three well‐characterized antibiotics whose mechanistic effects on bacterial cells are well‐studied at bulk and, to some extent, single‐cell level, but future research could expand to small‐population responses to other, less well understood, antibiotics or antibiotic combinations. However, we note that care should be taken to avoid leakage of antibiotics from the droplets, which would jeopardize the results of these experiments. The physicochemical properties of antibiotics, the concentrations used, and the distance between droplets can significantly influence leakage between droplets.^[^ ^67^ ^]^ Indeed, in early experiments in our lab with rifampicin, which is lipid soluble, we encountered issues with potential leakage. For the data presented here, antibiotic concentrations were low, and we did not observe any negative effects on the control population, confirming that leakage was not an issue. We also quantified the potential leakage of the antibiotics tested in our study, ruling out the possibility of any adverse effects caused by antibiotic leakage into droplets (Figure S31, Supporting Information).

Uncovering the underlying mechanisms behind the small‐population phenomena which we reveal here will require further research. It is already apparent, however, that systematic investigation of small‐population antibiotic response in microfluidic droplets could be relevant in both clinical and microbiological settings, since it provides insights that cannot be obtained from traditional bulk assays. This approach could lead the way to better understanding how antibiotics act on infections in complex, spatially partitioned geometries. This, in turn, may ultimately inform the design of dosing regimens that are better targeted, e.g., for early‐stage infections with small populations, or situations where infections are spatially fragmented, which could improve treatment efficacy and minimize the evolution of antibiotic resistance.

It should be noted that the droplet occupancy used in this work is governed by the Poisson distribution. In our experiments, droplets are loaded with an average of 8 cells per droplet, calculated using the average droplet size after generation and the initial loading cell density (Figure S1, Supporting Information). For this average occupancy, the Poisson probability that droplets will contain 4–12 cells is about 88%. Many droplets are predicted to have either lower or higher occupancy than the mean of 8 cells, suggesting that variation in population size is likely to be a significant factor in our results. In our investigation, we did not track individual droplets over the 24 h of the incubation period; therefore, with this methodology, we cannot link the initial occupancy of a given droplet to the observed outcome. In future, it would be very interesting to distinguish the effects of population size variability versus heterogeneity among isogenic bacterial cells in the population—both of which are present in these experiments. To disentangle these sources of variation, in future we plan to extend our studies with high‐throughput experiments in droplets, varying the population density from single‐cell inoculation to more dense populations. Although we focused here on the use of our multiplexed combinatorial droplet setup to study the antibiotic response of single‐species bacterial populations, droplet‐based combinatorial assays may also have broader applications, e.g., for understanding the dynamics of complex multispecies microbial communities in partitioned habitats.

## 4 Experimental Section

4.1

4.1.1

##### Bacterial Strain

The *E. coli* K12 strain RJA002^[^ ^68^ ^]^ was used in this study. RJA002 is a derivative of the MG1655 laboratory strain that contains a constitutively expressed yellow fluorescent protein (YFP) gene together with a chloramphenicol resistance marker. RJA002 was originally created by P1 transduction from strain MRR supplied by the Elowitz lab.^[^ ^69^ ^]^

Bacterial cells from an overnight culture were streaked onto an M9 agar plate and incubated for 24 h to obtain single bacterial colonies. For the microtiter plate and combinatorial droplet assay, a single colony from this plate was inoculated in M9 liquid media as the seed culture. As the main culture, an initial OD600 of 0.1 was prepared and incubated at 37 °C for around 2 h to reach an OD600 of 0.5. Aliquots of the main culture with an OD600 of 0.05 were used to start the microtiter plate and droplet assays.

##### Growth Media and Antibiotics

Droplet and bulk assays were done using M9 minimal medium at 37 °C. The prepared M9 medium components include 5X salt solution (Sigma‐Aldrich, 1:5 v v^−1^), 1 M MgSO_4_ (1:500 v v^−1^), 1 M CaCl_2_ (1:10 000 v v^−1^), and 0.4% glucose. Antibiotic stock solutions were prepared from frozen stocks. For the multiplexed droplet experiment, 8, 5, and 100 μg mL^−1^ stocks were prepared for tetracycline, streptomycin, and ampicillin, respectively, in the M9 medium for droplet generation.

##### Droplet Library Coding

Alexa‐Fluor 647 (Thermo Fischer), Cascade‐blue (Thermo Fischer), and DY557 (Dyomics) were used for color‐coding purposes. Two final stock concentrations of 6.4 and 32 μg mL^−1^ for Alexa‐Fluor 647 (Thermo Fischer) and 0.8 and 4.4 mg mL^−1^ for DY557 (Dyomics) were utilized for premixing in growth media, cell, and antibiotic solutions. A final concentration of 40 mg mL^−1^ was used to premix Cascade‐blue (Thermo Fischer). The dye‐mixed solutions are then loaded into the sample well stripe of the multiplexing platform. The multiplexing platform utilizes a commercial liquid handler, MitosDropix (Dolomite), that enables the precise production of reagent volumes down to 25 nL. Using the dedicated software for sample generation using MitosDropix (Dolomite), the multiplexed samples are produced and coded based on different portions of dyes in the final multiplexed sample. Various reagents are loaded into designated reservoirs of the device, as shown in Figure 1 and S1, Supporting Information. Using a sample hook, desired volumes of liquids (plugs) are withdrawn and merged in a transition from narrow to wider tubing in the platform (Figure 1 and S1, Supporting Information). The final multiplexed samples are then well‐mixed by oscillatory motion created by the syringe pump to produce uniform droplet populations. Finally, the samples are sent for droplet generation, and all droplets of different experimental conditions are collected.^[^ ^43^ ^]^ About 45 000 droplets were collected for every experimental condition. We avoided using a green coding dye in this experiment to eliminate the contamination in our assays, as our model *E. coli* strain produces YFP fluorescence.

##### Microscopy

The droplet library was imaged after generation (0 h) and after 8 and 24 h of incubation in an observation chamber. An inverted microscope (Axio Observer Z1, Zeiss) was used for image acquisition in five channels. To aid image analysis, brightfield images were taken with a numerical aperture of 0.16 to intensify cell edges.^[^ ^43^ ^]^ Fluorescence images were taken employing the Colibri 5 Zeiss microscope, and all images were taken with a 10× magnification. 16‐bit images in TIF format were saved for image analysis.

##### Image Analysis Pipeline and Machine Learning Decoding of Experimental Conditions

Python was used to create the image analysis and machine‐learning decoding approaches. Images of brightfield, YFP, red, far‐red, and blue channels in TIF format were fed into a custom Python script. Individual droplets were identified using the HoughCircles function of the OpenCV library. The average fluorescence intensity of color‐coding channels within every image was calculated using the information on droplets. A Gaussian blur function is first applied for brightfield growth analysis, followed by intensity rescaling to improve the image contrast of single droplets. Next, a Canny edge detection is applied to the region of interest within droplets. Finally, a morphology closing operation followed by dilation is performed, and the number of white pixels in the final binary image is counted and normalized to the area of the region of interest (proportional to droplet diameter) to quantify the growth within droplets. For YFP image analysis, the Sci‐Kit Learn ndimage package is used. 16‐bit YFP images are binarized by dynamic thresholding for every droplet, and connected components within the binary image are labeled as distinct objects. For each object, the size and aspect ratio are recorded for filamentous cell identification. The YFP object count is then calculated for the YFP cell distributions. To identify filamentous cells within droplets, aspect ratios of all objects in all droplets from 8 and 24 h of biological replicates 1 and 2 were utilized individually for K‐means clustering. Using the K‐means and setting the cluster number to two, we calculated the center point of clusters for the four K‐means runs. The average of these values was set as the aspect ratio threshold for filament identification (=2.5). Long filaments can be reliably identified with the area threshold of 200, which is manually chosen. All this information is saved for later analysis using machine‐learning algorithms. The zero‐hour images of droplets were employed for the brightfield thresholding used for plots. The average growth of the control population (no antibiotics) was calculated, and the threshold was set at the average plus two times the standard deviation. For the YFP analysis plots, the threshold was set at zero.

To categorize the experimental conditions of the mixed droplets, we have combined supervised classification with unsupervised clustering to analyze the data robustly. Using the droplet library's data right after production as the training data, we developed a machine‐learning algorithm based on a K‐neighbors classifier to identify different color combinations used within the droplet library. Then, for each color‐code combination, we employed hierarchical density‐based spatial clustering of applications with noise (HDBSCAN) to identify the color codes within each color combination. The individual color codes are then labeled with the corresponding antibiotic and concentration using a reference file of the color codes used in the experiment. This reference file is produced using the data from the droplet library after generation, and the experimental design of choosing the color codes for certain experimental conditions.

##### Antibiotic Leakage Evaluation

Droplets were prepared under the following four conditions and incubated separately in 2 mL tubes (Eppendorf) at 37 °C. Condition 1: droplets with the model bacterial strain, condition 2‐4: droplets with the model strain mixed with droplets of the maximum concentration of the antibiotics used in this study that are color‐coded with DY557 (Dyomics). Droplets containing bacterial cells were inoculated at an OD_600_ of 0.1, yielding an average of 14 cells per droplet with a droplet diameter of ≈70 μm. Droplets of each condition are imaged after generation and 24 h of incubation. Brightfield images from the control condition at 0 h are used to define a threshold for identifying droplets with increased biomass after 24 h, from where the droplet fraction with growth and average growth are calculated. The threshold is defined as the average growth from the 0‐hour brightfield image analysis of the control condition plus two standard deviations (=22.56).

##### Statistical Analysis

Python is used for all statistical analysis and data presentation. For the data analysis of the bulk antibiotic susceptibility assay, there are two technical replicates per concentration of antibiotics. For every concentration, the data points are corrected by the average of the first time‐point measurement of that concentration. The corrected data is then used for the line plot using Python, and the *y*‐axis demonstrates the relative biomass change. The sample size (i.e., the number of droplets) for each experimental condition is shown in Table S1–S3, Supporting Information. To evaluate the mean and standard deviation of the fraction of metabolically active droplets and of droplets showing growth above the brightfield threshold, the sample for each experimental condition is randomly divided into three subsets, and then the mean and standard deviation are calculated across the three subsets.

## Supporting Information

Supporting Information is available from the Wiley Online Library or from the author.

## Conflict of Interest

The authors declare no conflict of interest.

## Supporting information

Supplementary Material

## Data Availability

The data that support the findings of this study are available from the corresponding author upon reasonable request.
