# Supplementary material for: Probing Antibiotic Inhibition in Small Bacterial Populations With Combinatorial Droplet Microfluidics
Source: Small Sci. 2026 Jan 24;6(1):e202500421. doi: 10.1002/smsc.202500421 (PMC12849860; doi:10.1002/smsc.202500421)
Supplement: Supplementary file 1 — Supplementary Material [file SMSC-6-e202500421-s001.pdf]

Supporting Information

Probing antibiotic inhibition in small bacterial populations with combinatorial droplet microfluidics

Ashkan Samimi[a,b], Nia Verdon[c], Rosalind J. Allen[c,d], Miriam A. Rosenbaum[a,b,d]*

[a] *Leibniz Institute for Natural Product Research and Infection Biology – Hans-Knöll-Institute, Jena, Germany*
[b] *Faculty of Biological Sciences, Friedrich Schiller University, Jena, Germany*
[c] *Theoretical Microbial Ecology, Friedrich Schiller University, Jena, Germany*
[d] *Cluster of Excellence Balance of the Microverse, Friedrich Schiller University Jena, Jena, Germany*

[*]Corresponding author: Leibniz Institute for Natural Product Research and Infection Biology – Hans-Knöll-Institute, 07745 Jena, Germany

E-mail: miriam.rosenbaum@leibniz-hki.de (Miriam A. Rosenbaum)

Table of Contents

Supplementary tables 2

Sample sizes for different antibiotics 2

Supplementary figures 3

Estimated droplet occupancy 3

Combinatorial sample preparation platform 4

Correlation plots of YFP and brightfield analysis 5

Droplet images of the control population 17

Droplet images of the tetracycline populations 18

Fraction of filamentous cells for tetracycline-treated droplets 20

Tetracycline susceptibility assays for biological replicate 2 22

Fraction of filamentous cells for streptomycin-treated droplets 23

Droplet images of the streptomycin populations 24

Streptomycin susceptibility assays for biological replicate 2 27

Droplet images of the ampicillin populations 29

Ampicillin susceptibility assays for biological replicate 2 32

Brightfield growth analysis of ampicillin-treated droplets 33

Antibiotic leakage evaluation 33

Supplementary tables

Sample sizes for different antibiotics

Table S1. Sample sizes (i.e., numbers of droplets analyzed) for different concentrations of tetracycline (in µg mL^{-1}) for both biological replicates at 8 and 24 hours of incubation. The table shows the data in (8h, 24h) format.

Concentration	0	0.125	0.2	0.275	0.35	0.425	0.5
**replicate 1**	(259, 422)	(168, 160)	(137, 287)	(127, 276)	(214, 439)	(253, 277)	(243, 341)
**replicate 2**	(358, 585)	(147, 139)	(221, 155)	(165, 219)	(313, 262)	(330, 387)	(269, 325)

Table S2. Sample sizes (i.e., numbers of droplets analyzed) for different concentrations of streptomycin (in µg mL^{-1}) for both biological replicates at 8 and 24 hours of incubation. The table shows the data in (8h, 24h) format.

Concentration	0	0.1	0.25	0.35	0.45	0.6	0.7
**replicate 1**	(259, 413)	(229, 313)	(248, 433)	(279, 413)	(273, 437)	(276, 392)	(244, 405)
**replicate 2**	(358, 585)	(344, 422)	(357, 432)	(307, 392)	(317, 421)	(319, 399)	(313, 359)

Table S3. Sample sizes (i.e., numbers of droplets analyzed) for different concentrations of ampicillin (in µg mL^{-1}) for both biological replicates at 8 and 24 hours of incubation. The table shows the data in (8h, 24h) format.

Concentration	0	5	10	13	17	21	25
**replicate 1**	(259, 422)	(177, 226)	(244, 345)	(277, 386)	(259, 413)	(212, 272)	(250, 377)
**replicate 2**	(358, 585)	(176, 320)	(334, 354)	(108, 47)	(344, 346)	(302, 402)	(300, 205)

Supplementary figures

Estimated droplet occupancy

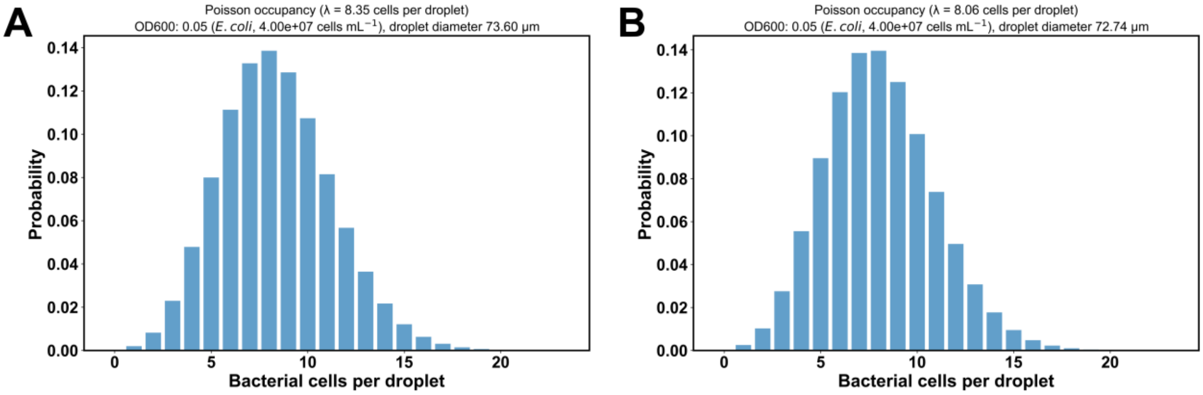

Figure S1. Estimated Poisson distribution of bacterial cell occupancy within droplets for biological replicate 1 (**A**) and replicate 2 (**B**). The average droplet diameter across all conditions was calculated at the 0-hour time point, and the theoretical distribution was estimated based on the initial loading cell density (OD_{600} = 0.05).

Combinatorial sample preparation platform

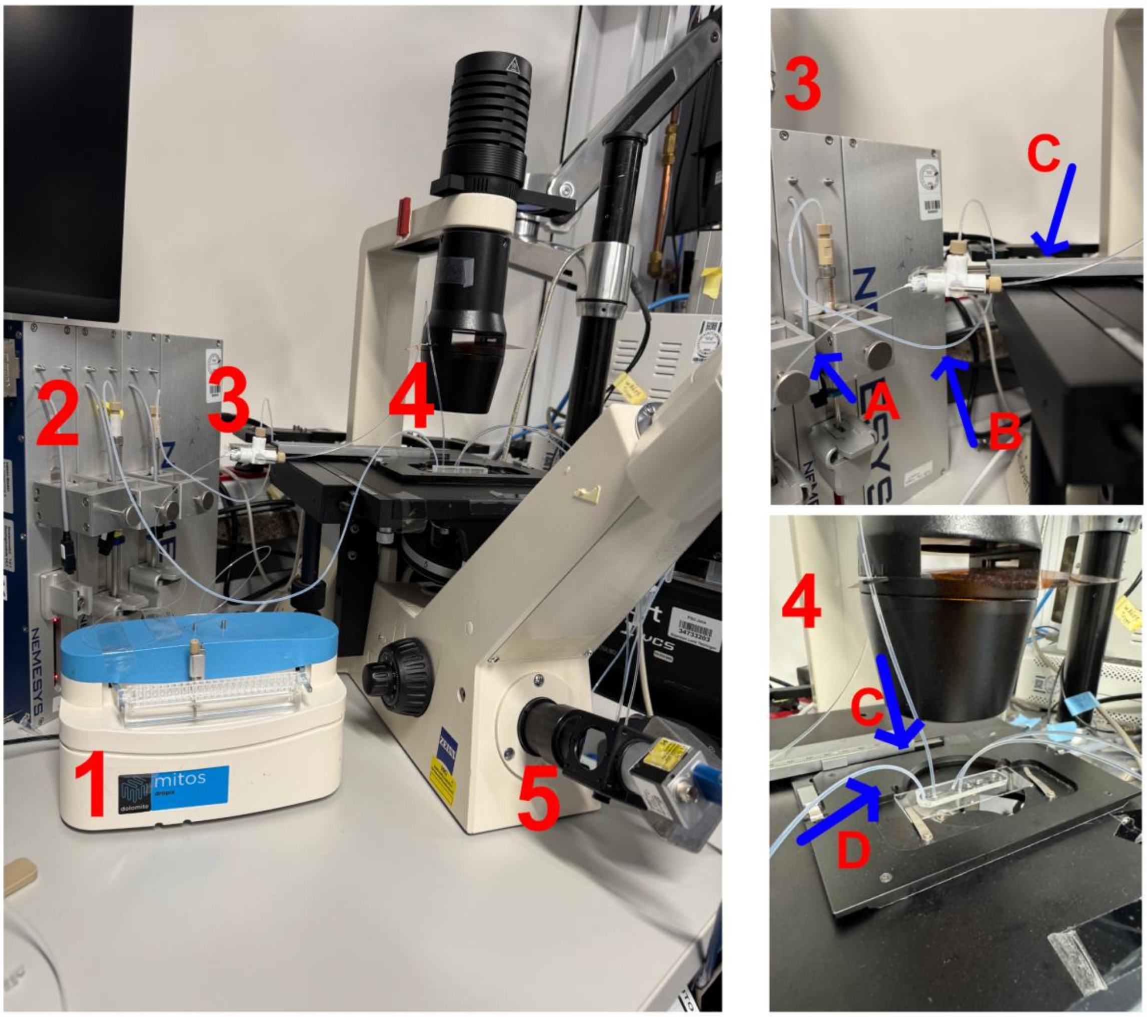

Figure S2. Photographs of the combinatorial sample preparation platform used for droplet assays in this study. The platform consists of five sections. In section 1, there is a commercial liquid handler called MitosDropix (Dolomite) with an oil reservoir and a 24-well strip for loading different reagents. As shown in the image, there is also a sample hook that moves between and within the wells to take a defined amount of liquid. Later, by merging different reagents, a multiplexed sample is created. The fluidic operations are controlled by a syringe pump displayed in stage 2 of the platform. Additionally, a manual switching one-to-two-way fluidic valve in stage 3 directs the fluid to different tubing, either for storage or for sending to droplet generation in stage 4. On the right side of the image, a detailed view of stages 3 and 4 is provided. At the start of sample preparation, tubing A (a narrow tube, 0.25 mm inner diameter, indicated by the blue arrow) and B (a wider tube, 1.5 mm inner diameter, indicated by the blue arrow) are connected through the manual valve. The small volumes of liquid from the MitosDropix merge during this fluidic transition when they are closely spaced, resulting in a multiplexed sample for droplet generation. The manual valve is then switched so that tubing B connects to C. At this point, the syringe pump is programmed to create oscillatory flow for proper mixing, producing a homogeneous solution for droplet formation. The samples are then sent to stage 4 for droplet generation. As shown, the samples from tubing C are chopped into picoliter droplets using oil from tubing D. At this stage, the outlet of the microfluidic chip is controlled by a valve to switch

between collecting droplets and dumping droplets to waste. A wash plug of phosphate-buffered saline is introduced between samples to clean the chip and avoid cross-contamination, and the outlet control only allows droplets produced from each sample to be collected. The switching of outlets is managed using the droplet counter in stage 5, which features an optical counter with a beam splitter that directs light to both an observation camera and a photodiode. This setup provides a real-time count of generated droplets, and along with the timing between samples (dictated by flow rates), the outlets are switched accordingly.

Correlation plots of YFP and brightfield analysis

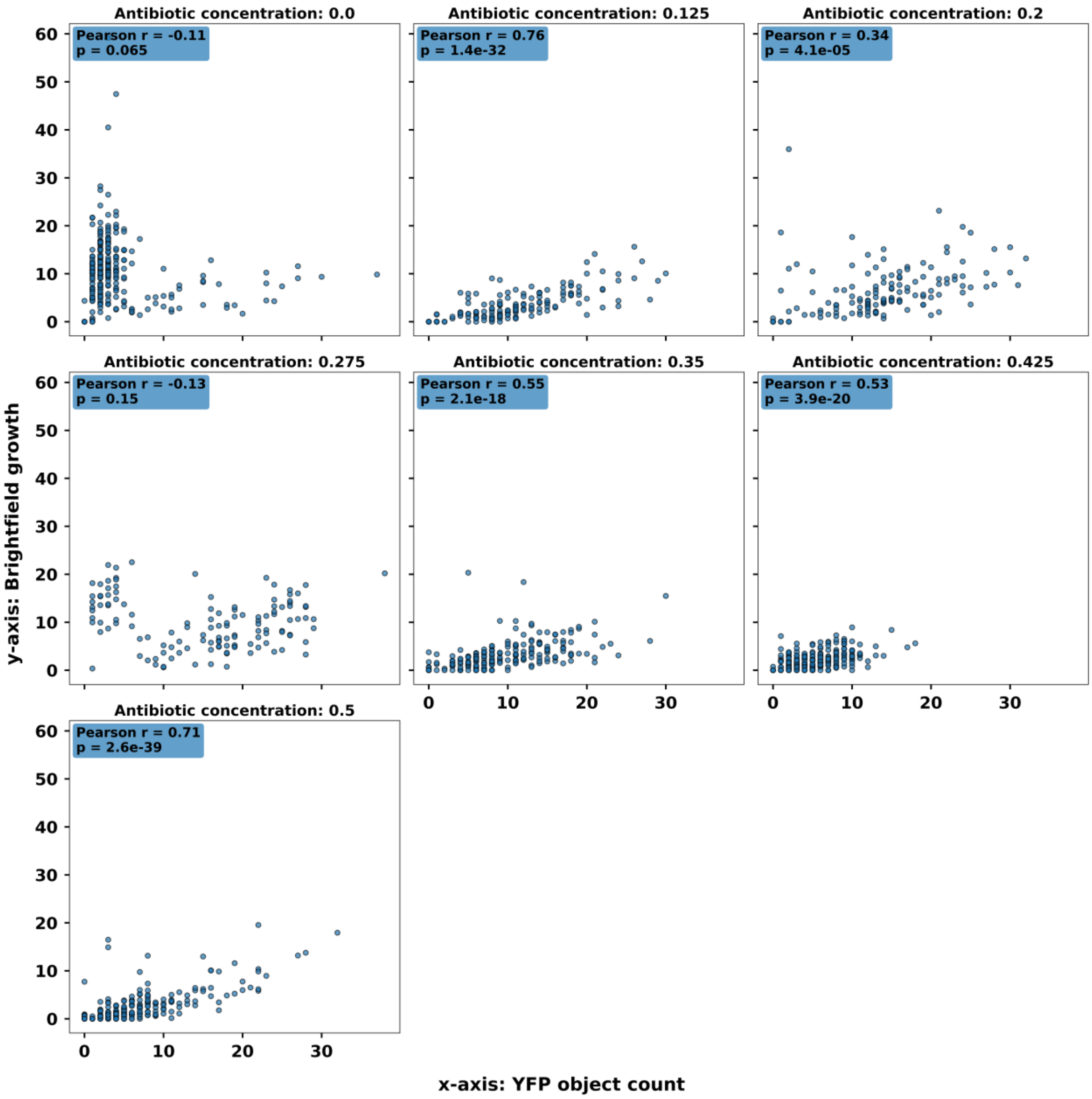

Figure S3. Correlation plot comparing YFP object count and brightfield growth for tetracycline after 8 hours of incubation for biological replicate 1. There is a positive correlation for most of the antibiotic conditions, with some variability. As shown in the plot, for the control and antibiotic concentrations of 0.275 µg mL^{-1}, there is no strong positive correlation. These variabilities originate from the image analysis logic for brightfield and YFP images, which measure different qualities of growth (see Materials and Methods for detailed explanations). In the YFP analysis, a large aggregation is counted as one object, whereas in the brightfield analysis, it covers a larger area, leading to a higher growth

value (one object count in YFP compared to a 10-30 pixel area quantified in brightfield image analysis). Also, in the YFP analysis, partly dispersed cells in each droplet result in more variation in the number of objects (i.e., the proxy for the number of bacterial cells). However, in brightfield analysis, the area is less sensitive to the number of dispersed cells, as they do not significantly change the area covered within droplets.

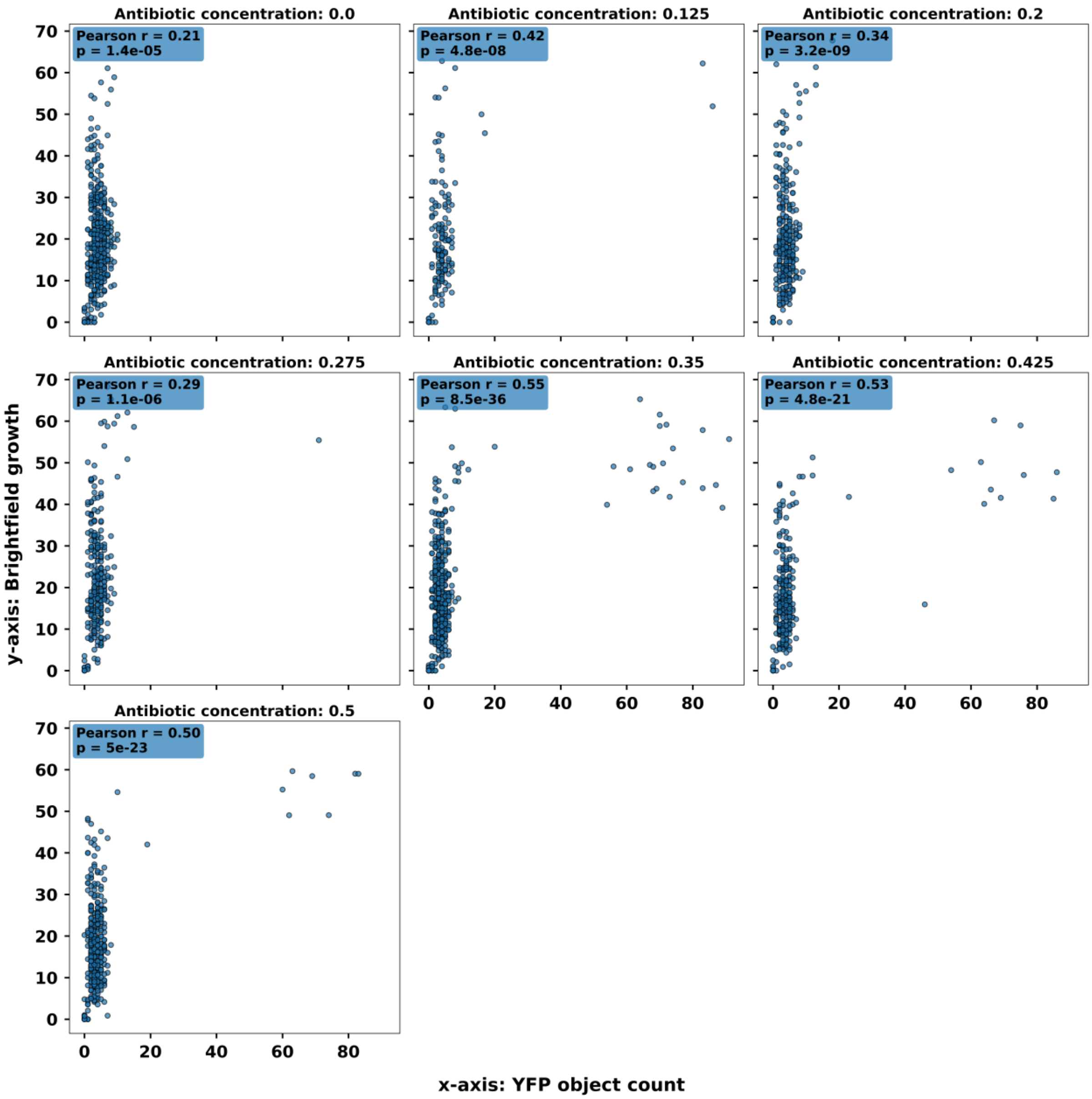

Figure S4. Correlation plot comparing YFP object count and brightfield growth for tetracycline after 24 hours of incubation for replicate 1. There is a positive correlation between the two analysis approaches, with some variability due to differences in the image analysis logic of brightfield and YFP images, as explained in Figure S2.

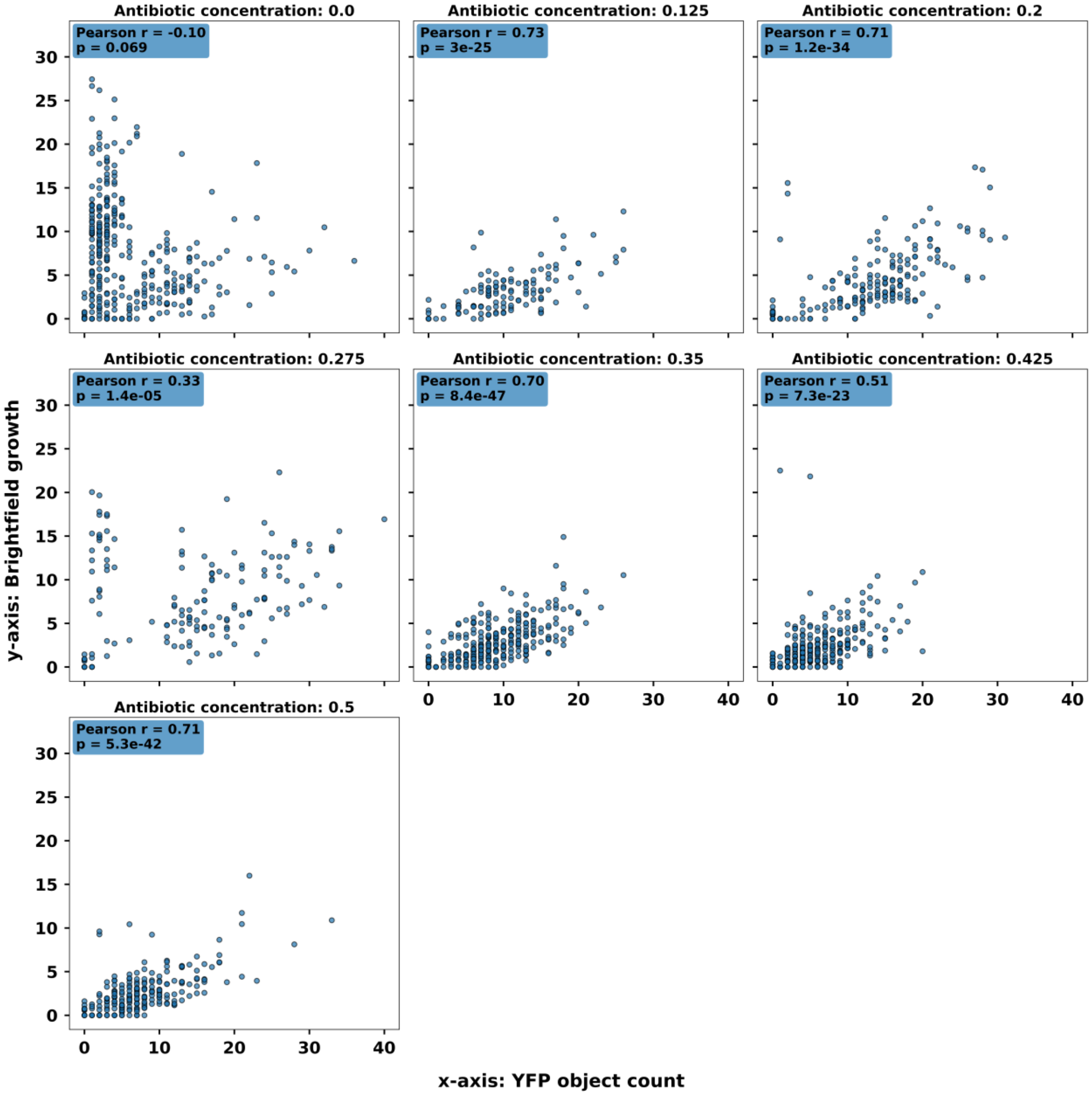

Figure S5. Correlation plot comparing YFP object count and brightfield growth for tetracycline after 8 hours of incubation for biological replicate 2. Similar results are observed as in replicate 1.

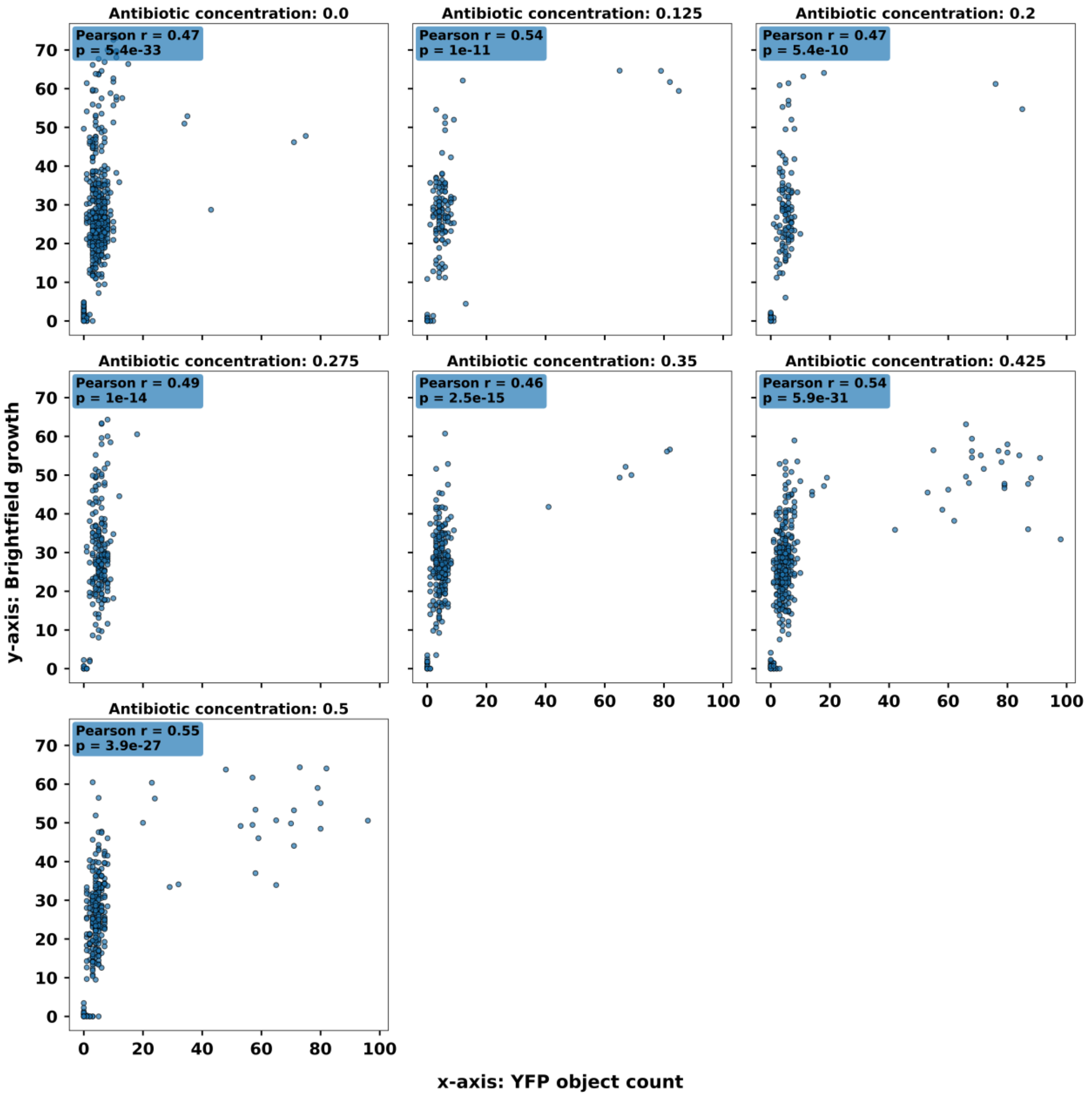

Figure S6. Correlation plot comparing YFP object count and brightfield growth for tetracycline after 24 hours of incubation for biological replicate 2. Similar results are observed as in biological replicate 1.

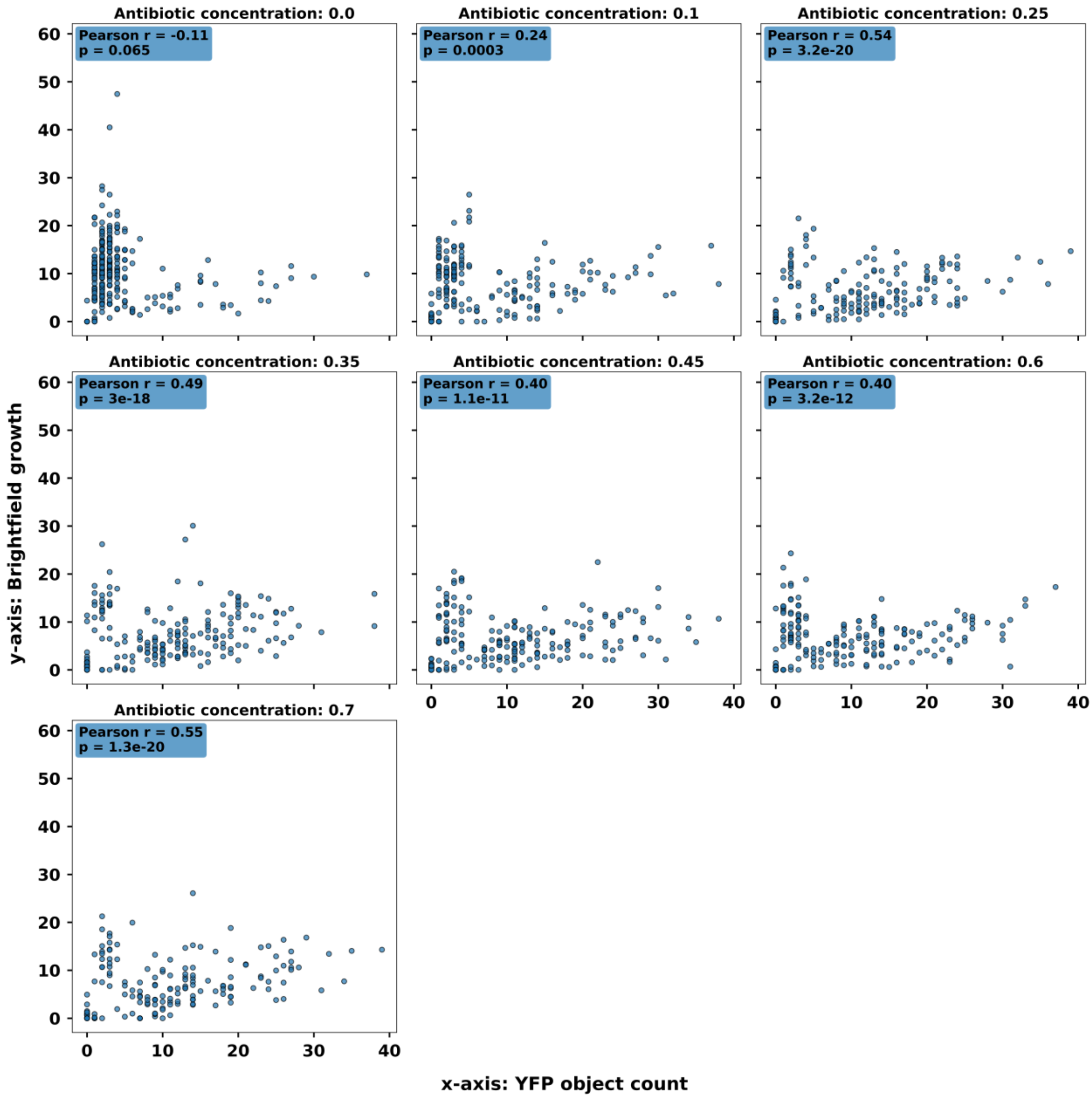

Figure S7. Correlation plot comparing YFP object count and brightfield growth for streptomycin after 8 hours of incubation for biological replicate 1. There is a positive correlation between the two analysis approaches, with some variability due to differences in the image analysis logic of brightfield and YFP images, as explained in Figure S2.

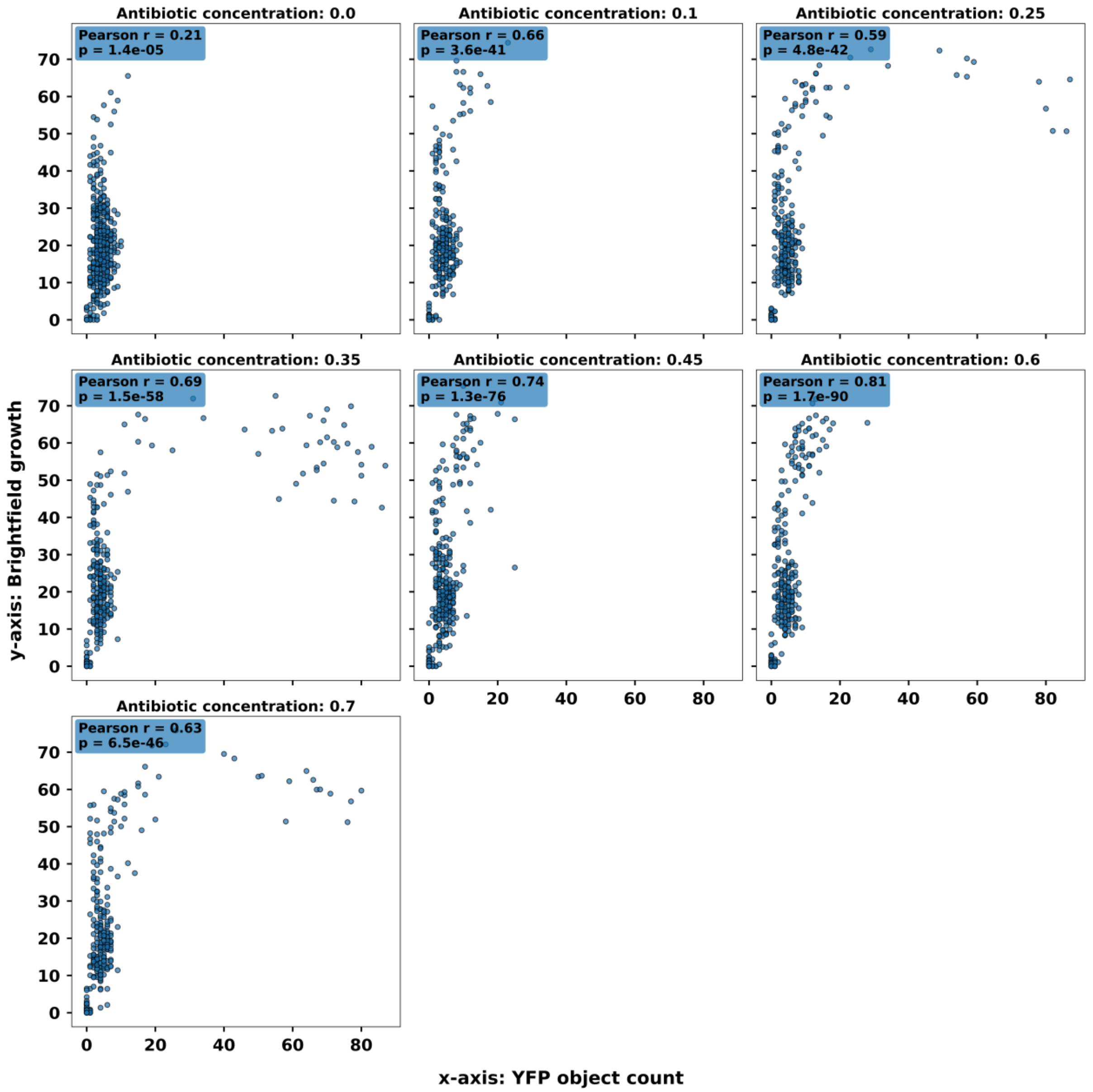

Figure S8. Correlation plot comparing YFP object count and brightfield growth for streptomycin after 24 hours of incubation for biological replicate 1. There is a positive correlation between the two analysis approaches, with some variability due to differences in the image analysis logic of brightfield and YFP images, as explained in Figure S2.

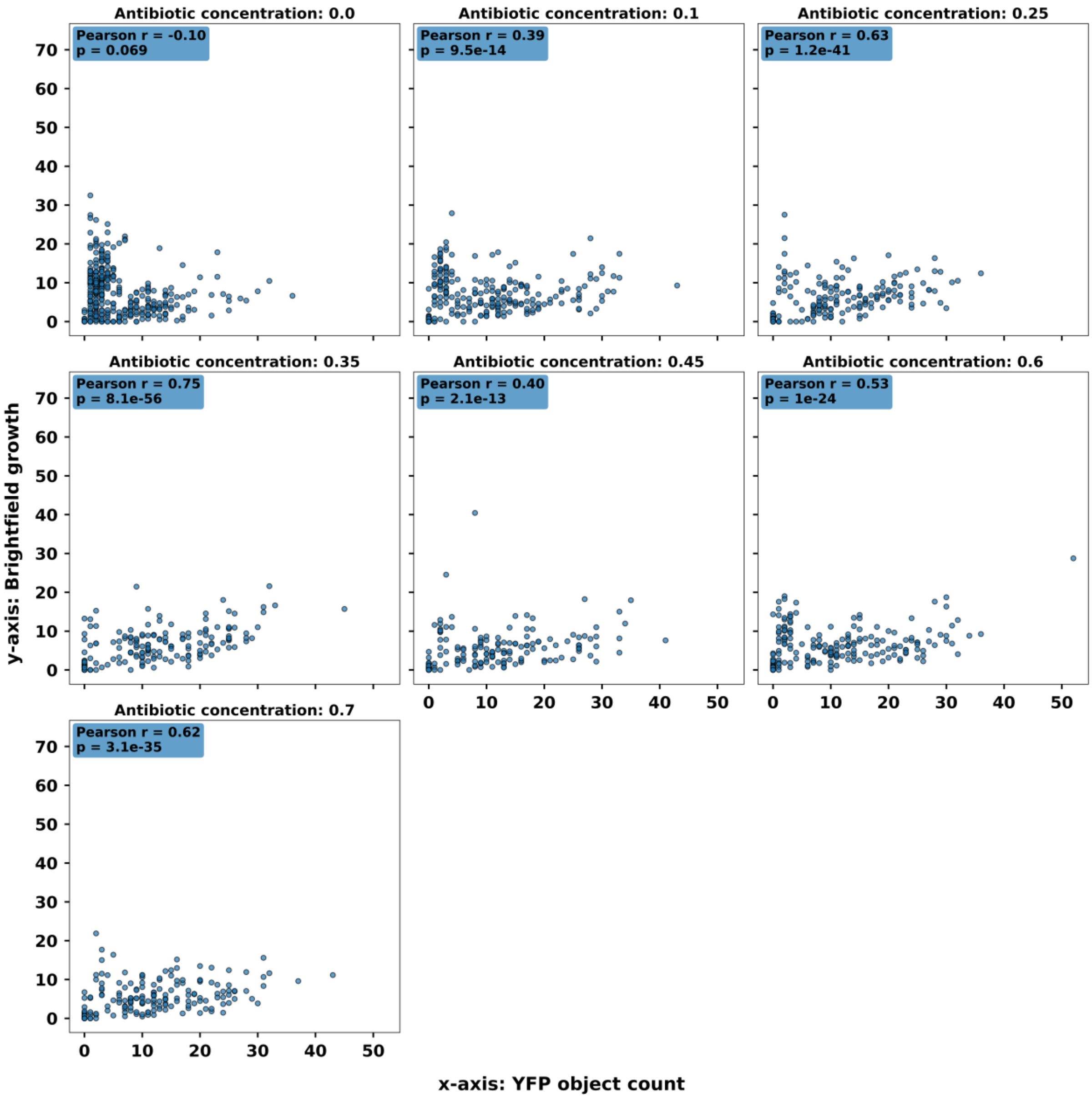

Figure S9. Correlation plot comparing YFP object count and brightfield growth for streptomycin after 8 hours of incubation for biological replicate 2. Similar results are observed as in biological replicate 1.

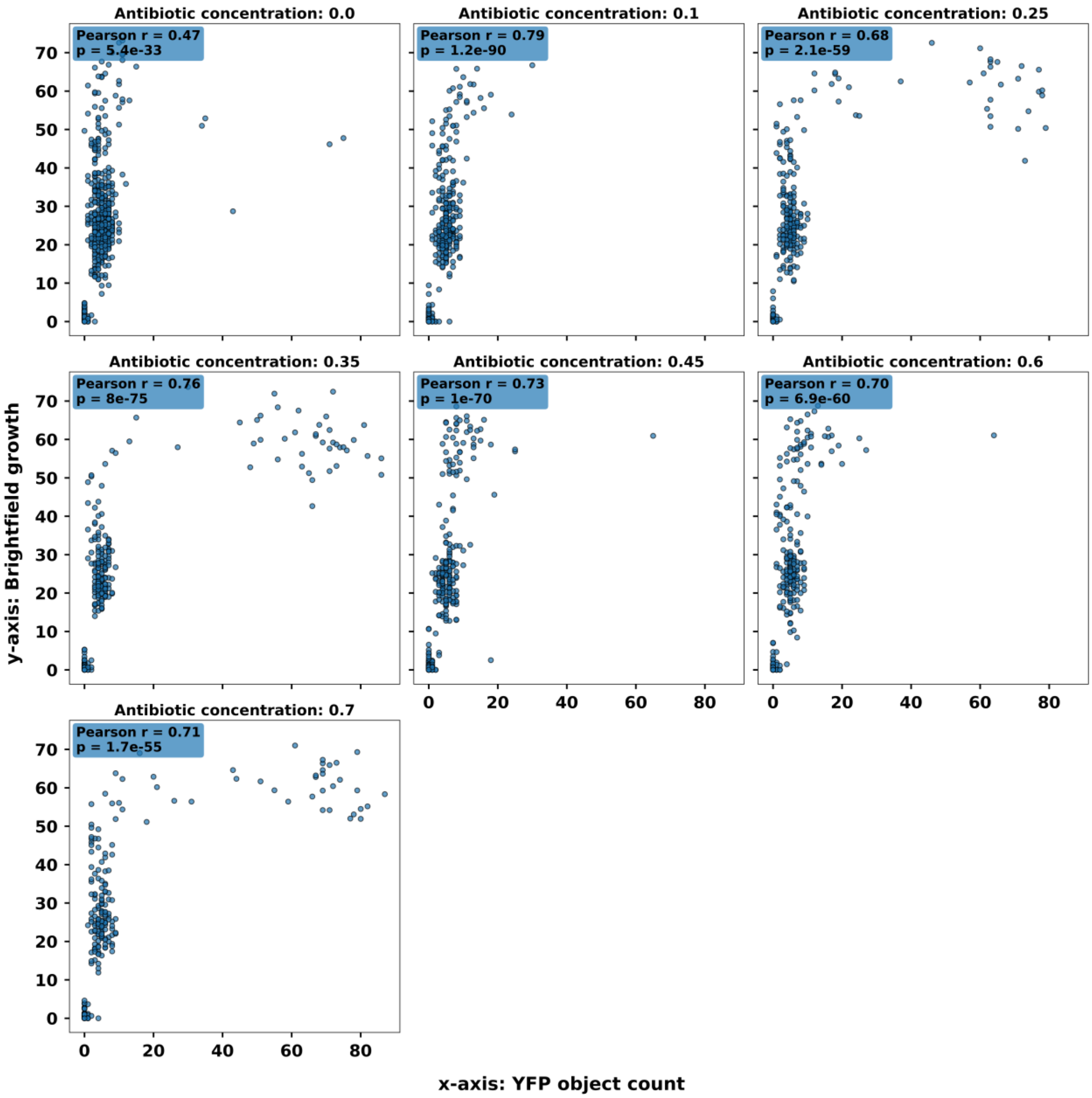

Figure S10. Correlation plot comparing YFP object count and brightfield growth for streptomycin after 24 hours of incubation for biological replicate 2. Similar results are observed as in biological replicate 1.

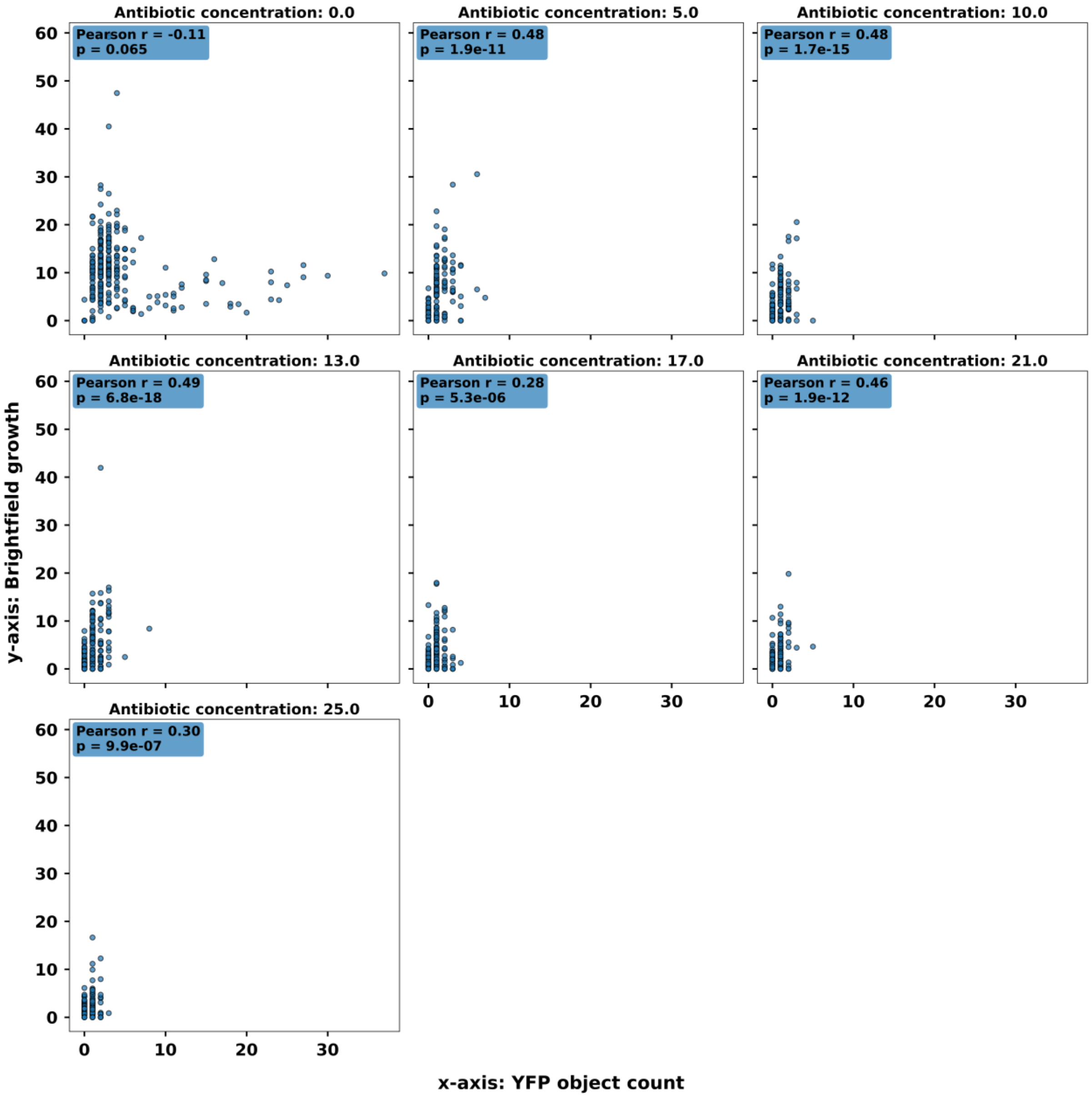

Figure S11. Correlation plot comparing YFP object count and brightfield growth for ampicillin after 8 hours of incubation for biological replicate 1. There is a positive correlation between the two analysis approaches, with some variability due to differences in the image analysis logic of brightfield and YFP images, as explained in Figure S2. However, the correlation between the two analysis approaches decreases in the case of ampicillin. During antibiotic-induced cell lysis, cell remnants are quantified as bacterial biomass through brightfield images, but there are few or no objects (i.e., bacterial cells) detected in YFP image analysis. This introduces variability between the analysis approaches, leading to reduced correlation between the two methods.

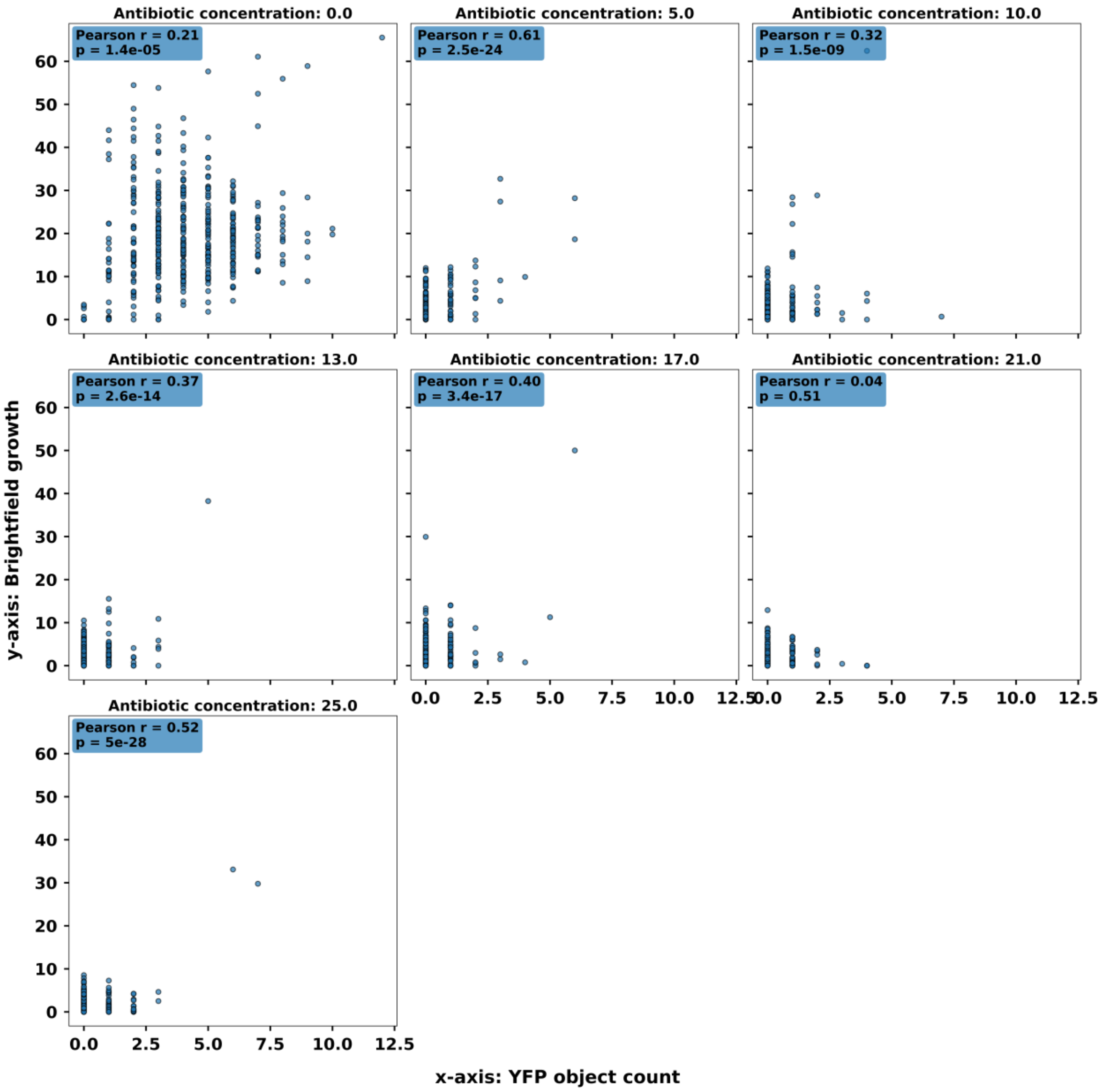

Figure S12. Correlation plot comparing YFP object count and brightfield growth for ampicillin after 24 hours of incubation for biological replicate 1. There is a positive correlation between the two analysis approaches, with some variability due to differences in the image analysis logic of brightfield and YFP images, as explained in Figure S2 and Figure S10.

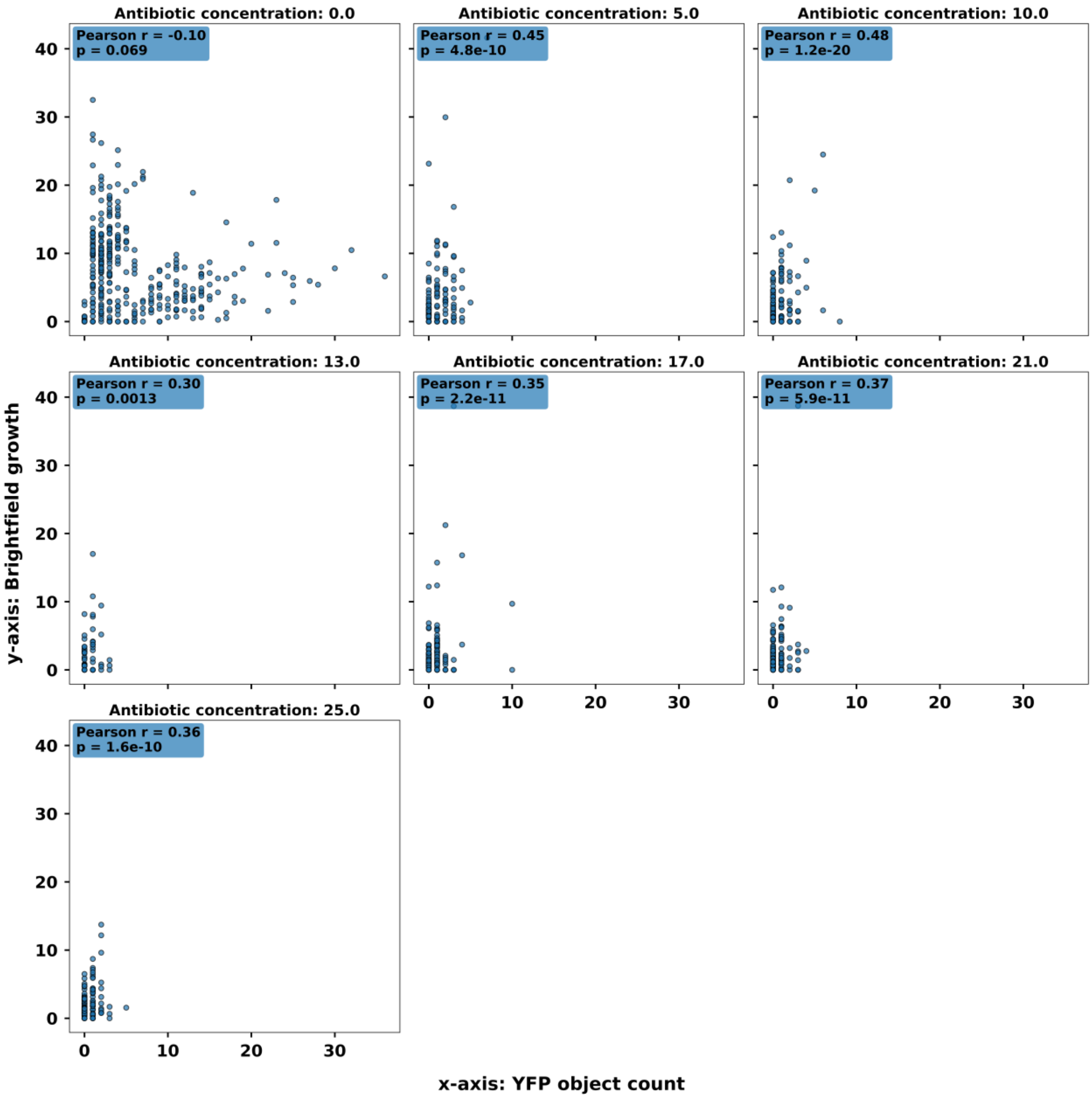

Figure S13. Correlation plot comparing YFP object count and brightfield growth for ampicillin after 8 hours of incubation for biological replicate 2. Similar results are observed as in biological replicate 1.

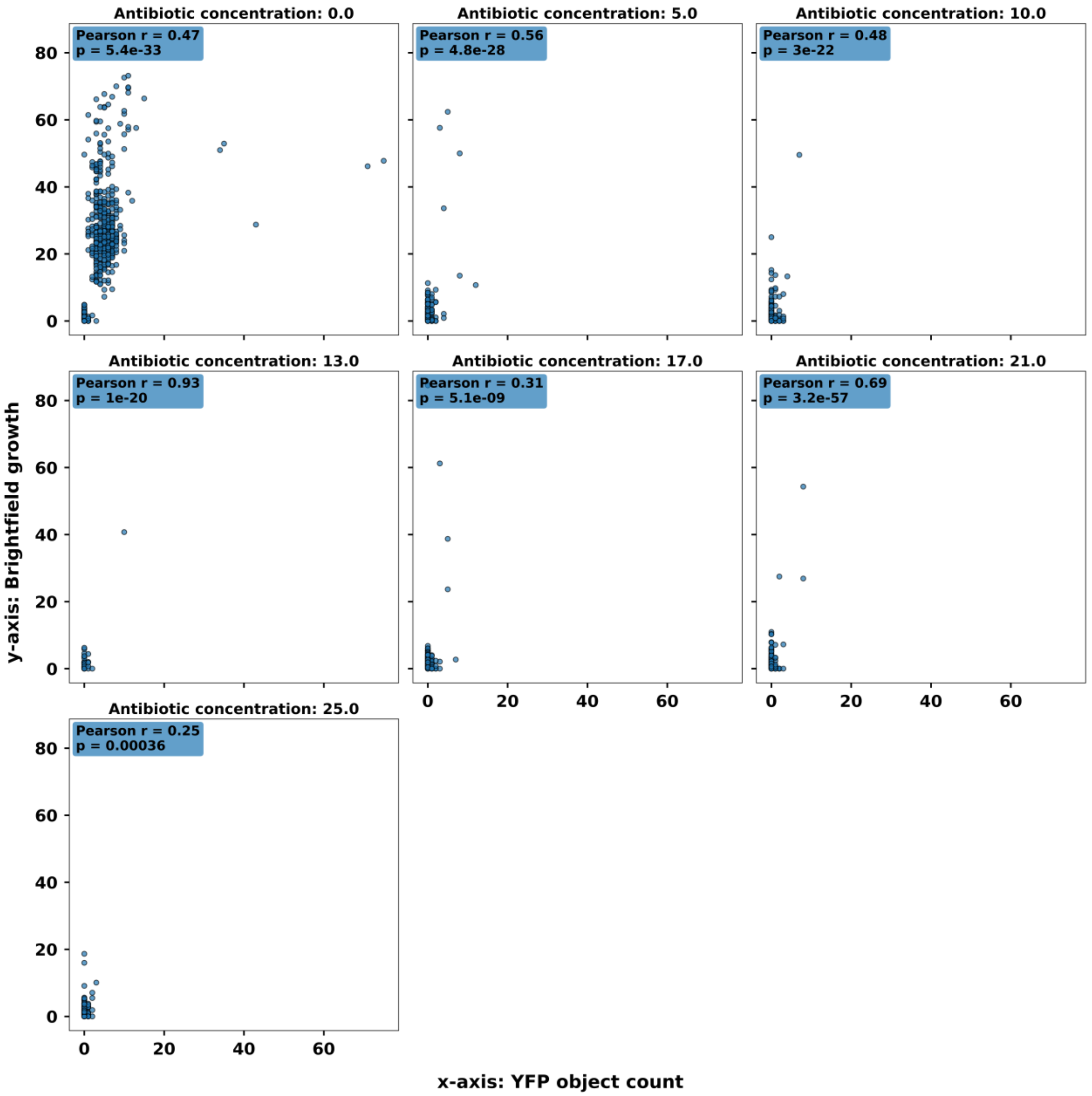

Figure S14. Correlation plot comparing YFP object count and brightfield growth for ampicillin after 24 hours of incubation for biological replicate 2. Similar results are observed as in biological replicate 1.

Droplet images of the control population

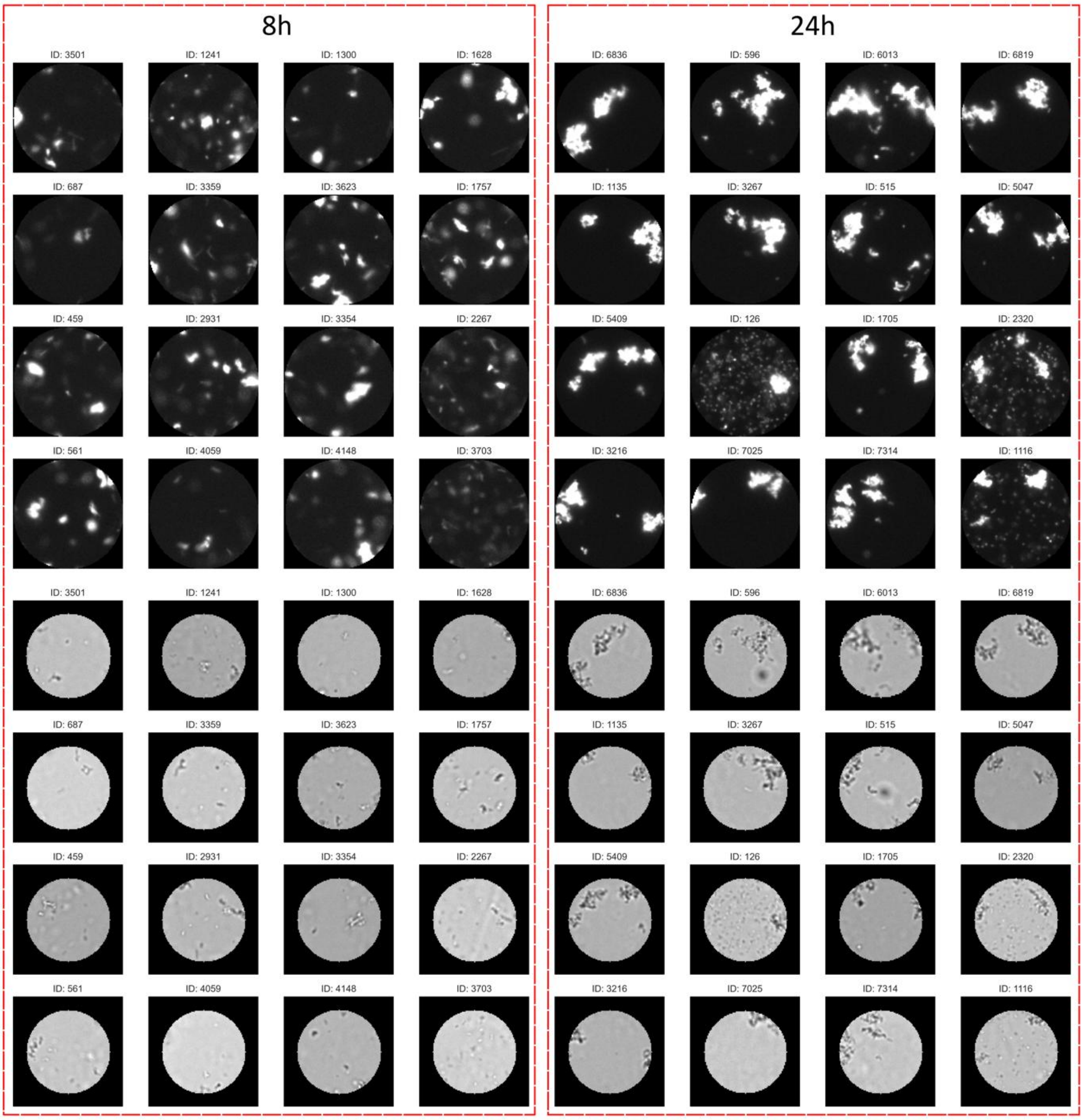

Figure S15. Droplet images for the no antibiotic control in the bright field (bottom rows) and YFP channels (top rows). Randomly selected droplets from biological replicate 1 are shown after 8 and 24 hours of incubation.

Droplet images of the tetracycline populations

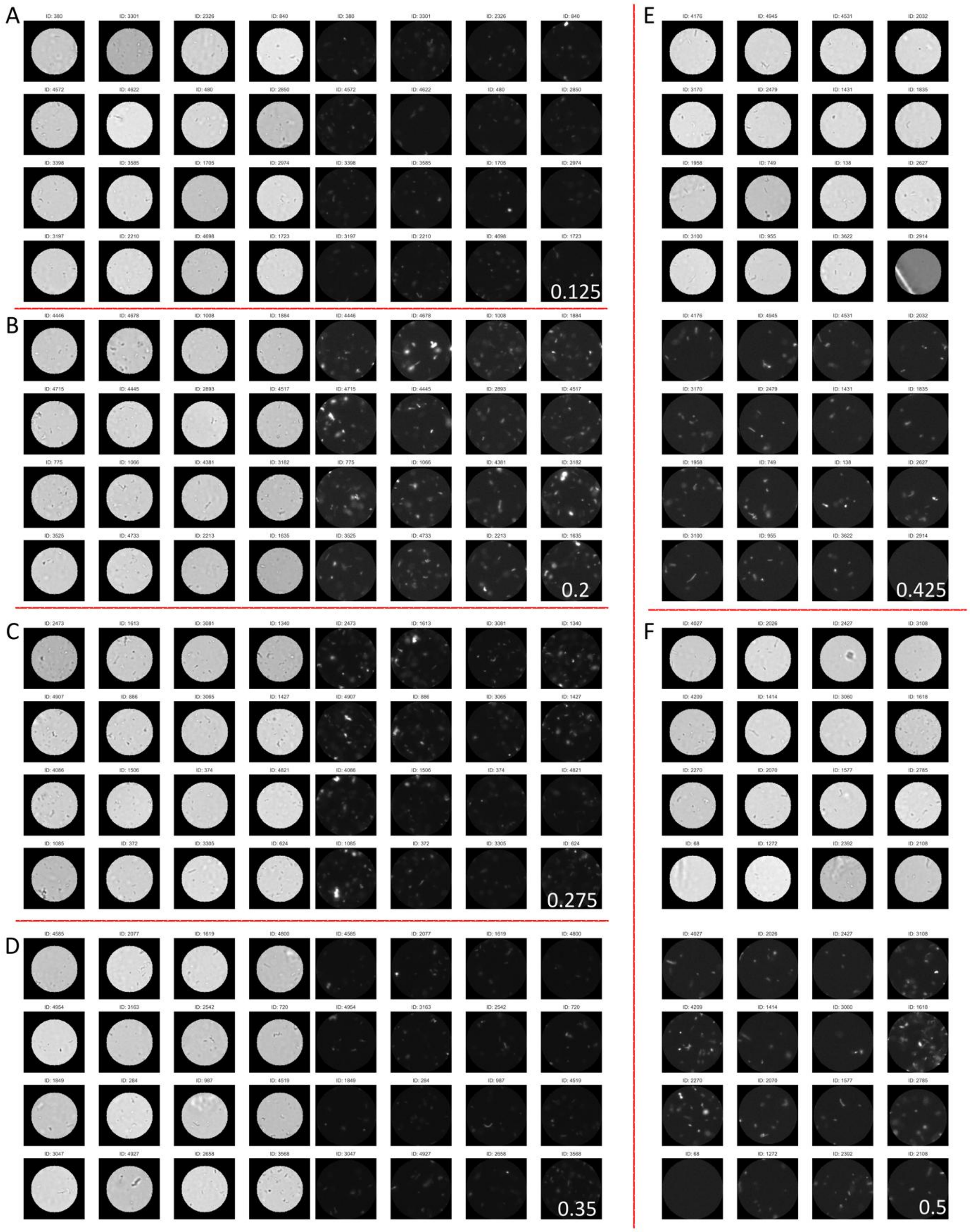

Figure S16. Droplet images corresponding to different tetracycline concentrations, after 8 hours of incubation, in the bright field and YFP channels. The droplet images are randomly chosen, but only droplets that exhibit non-zero growth (based on bright field growth data and thresholding conditions for each replicate; please check materials and methods) are shown. The numbers in each set of images indicate the antibiotic concentration in μg mL^{-1}units.

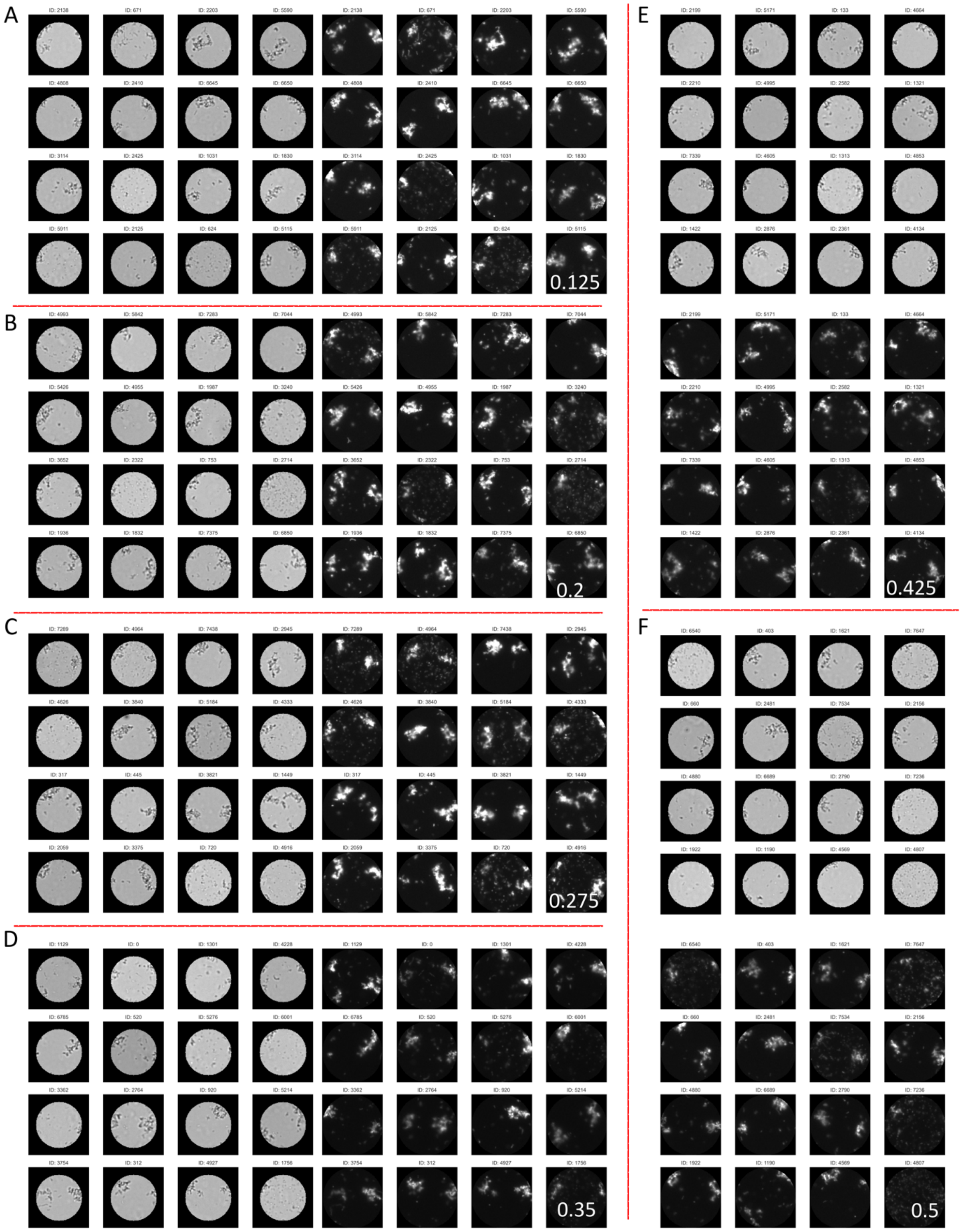

Figure S17. Droplet images corresponding to different tetracycline concentrations, after 24 hours of incubation, in the bright field and YFP channels. The droplet images are randomly chosen, but only droplets that show non-zero growth (based on bright field growth data and thresholding conditions for each replicate; please check materials and methods) are shown. The numbers in each set of images indicate the antibiotic concentration in μg mL^{-1}.

Fraction of filamentous cells for tetracycline-treated droplets

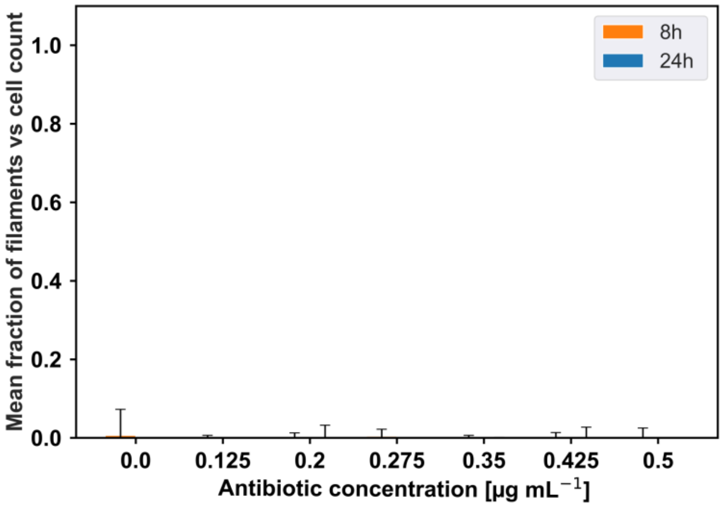

Figure S18. Fraction of filamentous cells within droplets for tetracycline-treated droplets corresponding to biological replicate 1. The aspect ratios of all objects (i.e., bacterial cells) in droplets are calculated, and a threshold is determined using K-means clustering to distinguish filamentous/non-filamentous bacterial cells. For the definition of the filamentous cell fraction, see materials and methods in the main text. The fraction of filamentous cells is very low at all the tested concentrations of tetracycline.

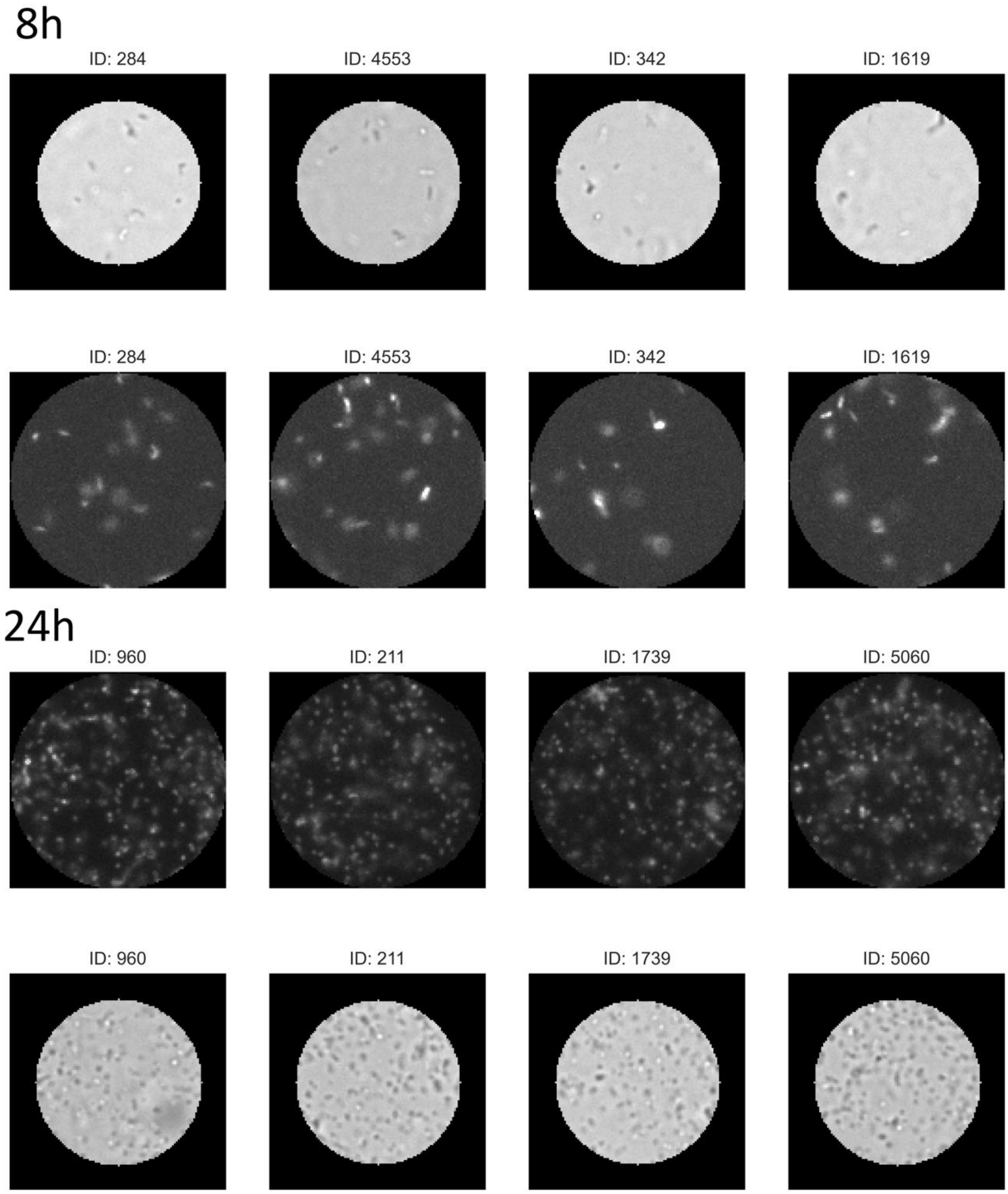

Figure S19. Example images showing selected droplets (with object counts above 10; i.e., the number of bacterial cells within every droplet) in the presence of tetracycline at 0.35 µg mL^{-1} after 8 hours incubation in YFP (lower images) and brightfield (upper images), and after 24 hours incubation in YFP (up) and brightfield (down). The images show multiple non-filamentous cells, indicating a dispersal growth pattern after 24 hours.

Tetracycline susceptibility assays for biological replicate 2

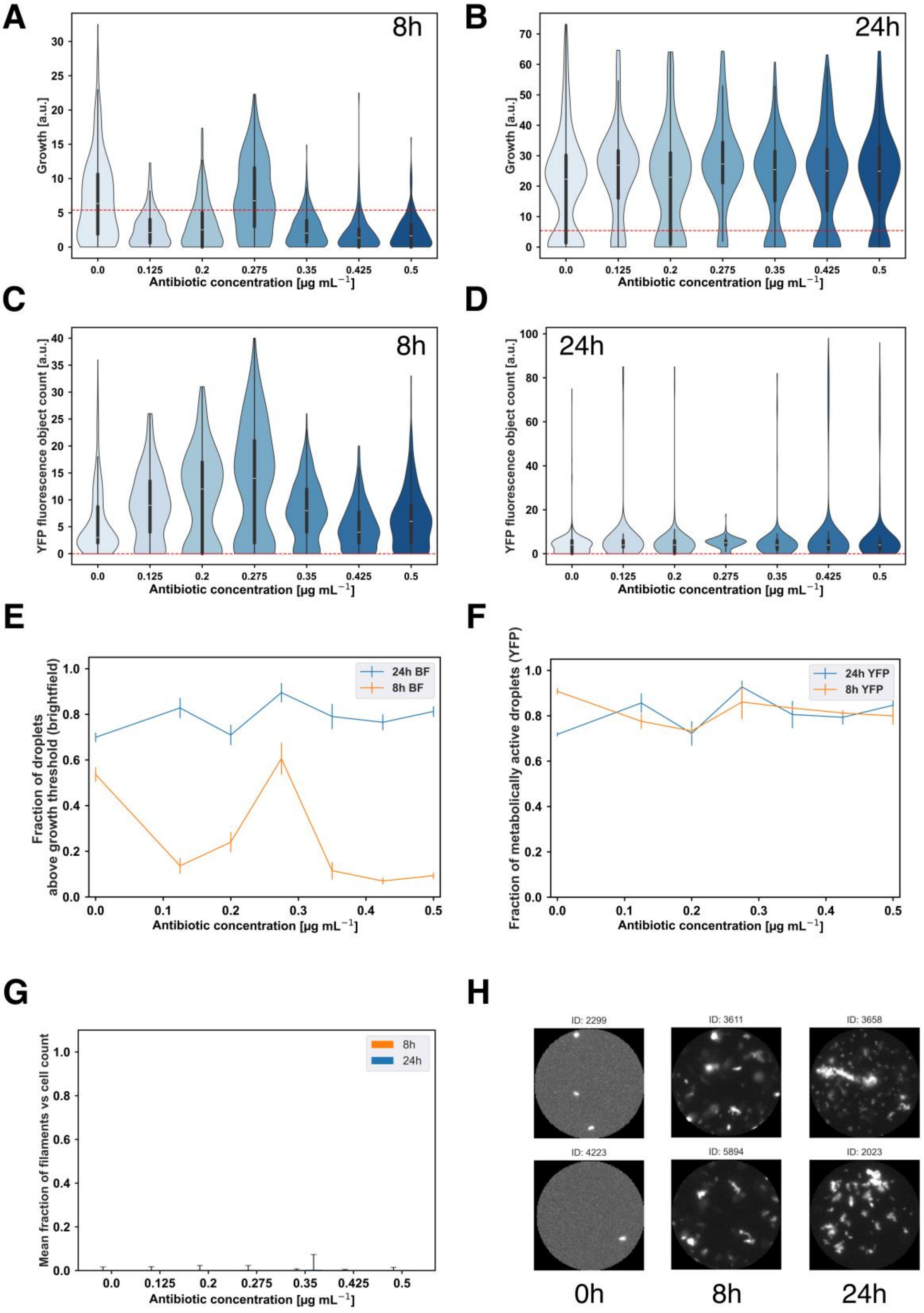

Figure S20. Tetracycline susceptibility assays for *E. coli* K12 strain RJA002, corresponding to biological replicate two. Droplet experiments were conducted in the sub-inhibitory range of 0.125 to 0.5 µg mL^{-1} tetracycline. Violin plots for the growth of droplets exposed to different tetracycline

concentrations are calculated by quantifying the growth within individual droplets utilizing bright field images after 8 (A) and 24 (B) hours of incubation. Using the bright field images of the 0-hour control population (no antibiotic), a threshold line (dashed red line) is defined (=3.4 and 5.4 for biological replicate 1 and 2, respectively) to distinguish empty and filled droplets at later time points for all antibiotic concentrations. (C) and (D) demonstrate the violin plot of object counts within droplets, demonstrating the cell number distribution. (E) shows the fraction of droplets exhibiting growth higher than the designated brightfield growth threshold. (F) shows the fraction of metabolically active droplets, defined as those whose YFP object count is greater than zero. Error bars indicate the standard deviation, calculated by randomly splitting the dataset for each antibiotic concentration into three sub-sets. The fraction of cells showing filamentous growth within individual droplets for different antibiotic concentrations is shown in (G). (H) shows randomly picked images of tetracycline droplets at 0.275 μg/ml at different time points. Minimum droplet number n = 139 and maximum n = 585.

Fraction of filamentous cells for streptomycin-treated droplets

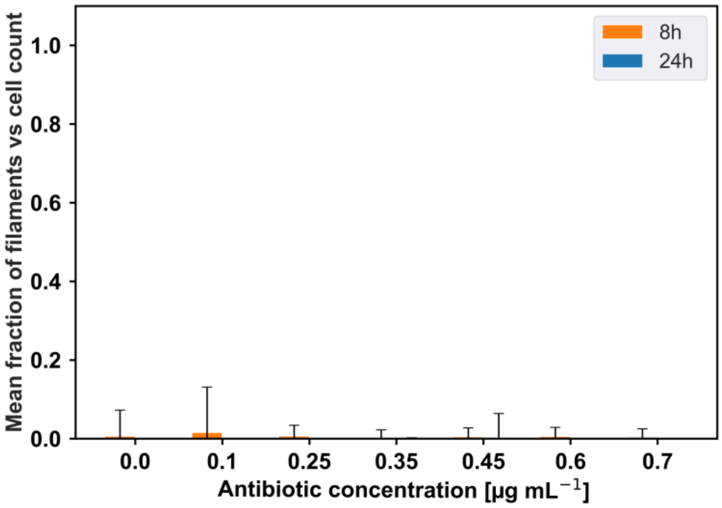

.

Figure S21. Fraction of filamentous cells within droplets for streptomycin-treated droplets corresponding to biological replicate 1. The aspect ratios of all objects (i.e., bacterial cells) in droplets are calculated, and a threshold is determined using K-means clustering to distinguish filamentous/non-filamentous bacterial cells. For the definition of the filamentous cell fraction, see materials and methods in the main text. The fraction of filamentous cells is very low at all the tested concentrations of streptomycin. Minimum droplet number n = 229 and maximum n = 437.

Droplet images of the streptomycin populations

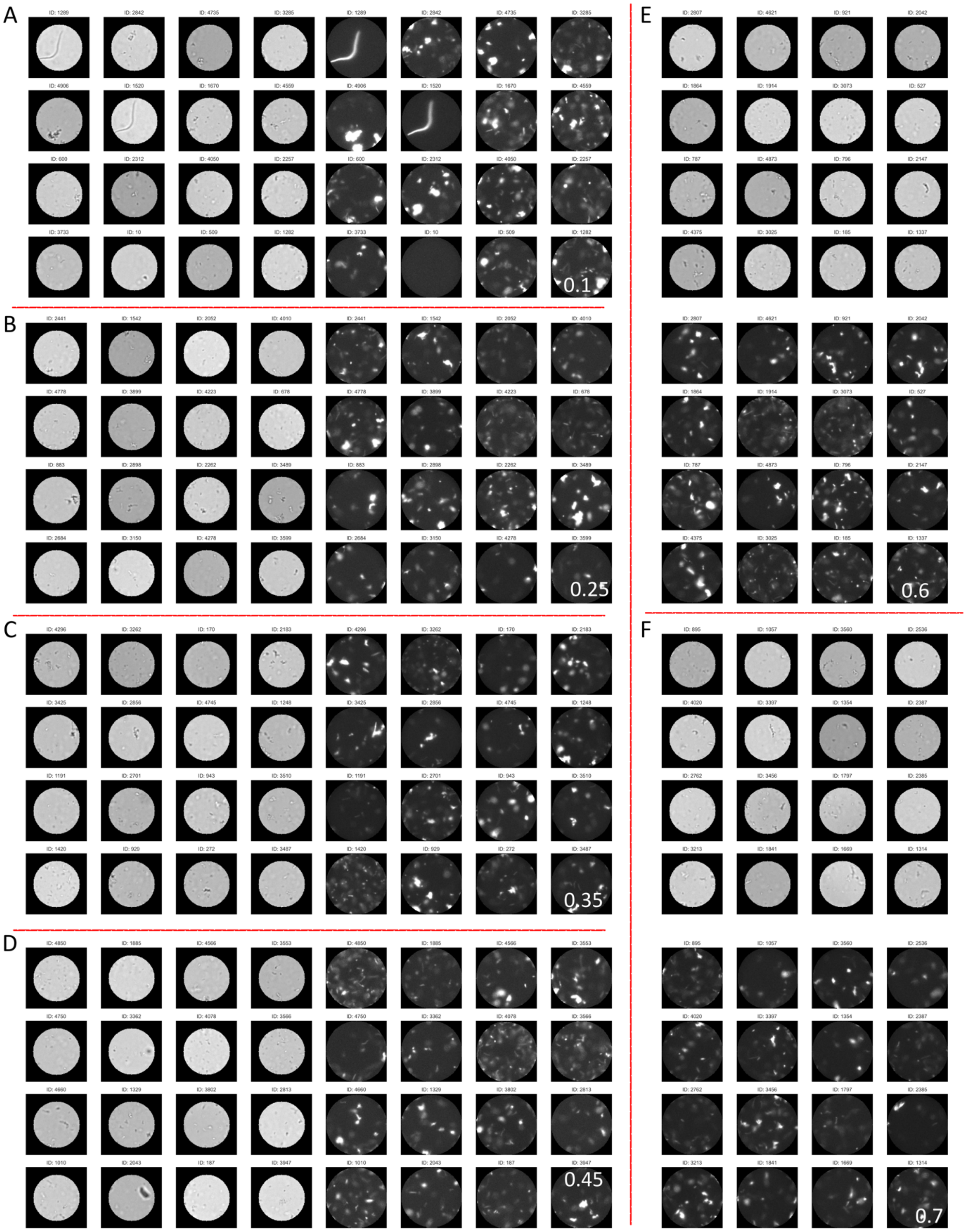

Figure S22. Droplet images corresponding to different streptomycin concentrations, after 8 hours of incubation, in the bright field and YFP channels. The droplet images are randomly chosen, but only droplets that exhibit non-zero growth (based on bright field growth data and thresholding conditions for each replicate; please check materials and methods) are shown. The numbers in each set of images indicate the antibiotic concentration in µg mL^{-1}.

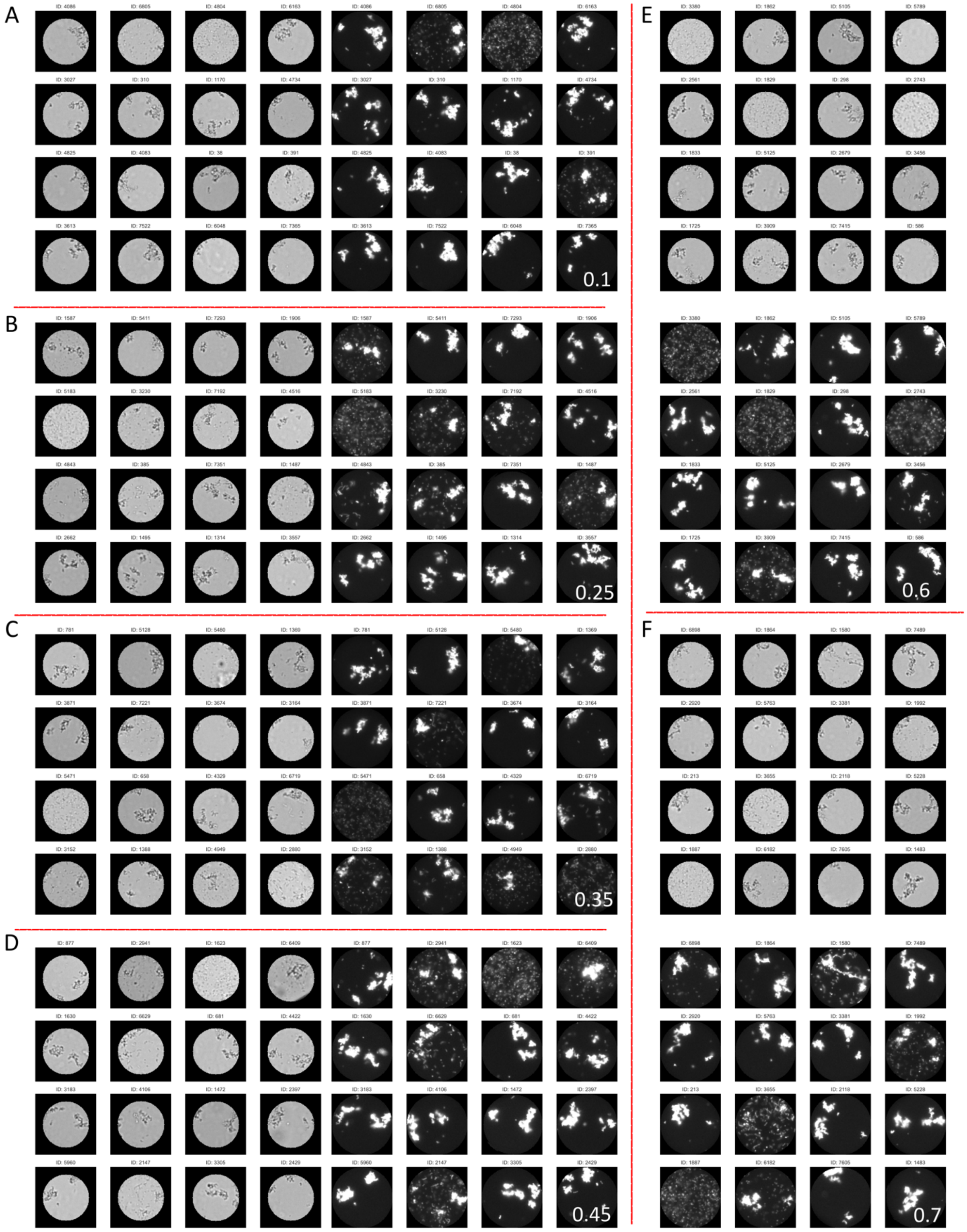

Figure S23. Droplet images corresponding to different streptomycin concentrations, after 24 hours of incubation, in the bright field and YFP channels. The droplet images are randomly chosen, but only droplets that exhibit non-zero growth (based on bright field growth data and thresholding conditions for each replicate; please check materials and methods) are shown. The numbers in each set of images indicate the antibiotic concentration in μg mL^{-1}.

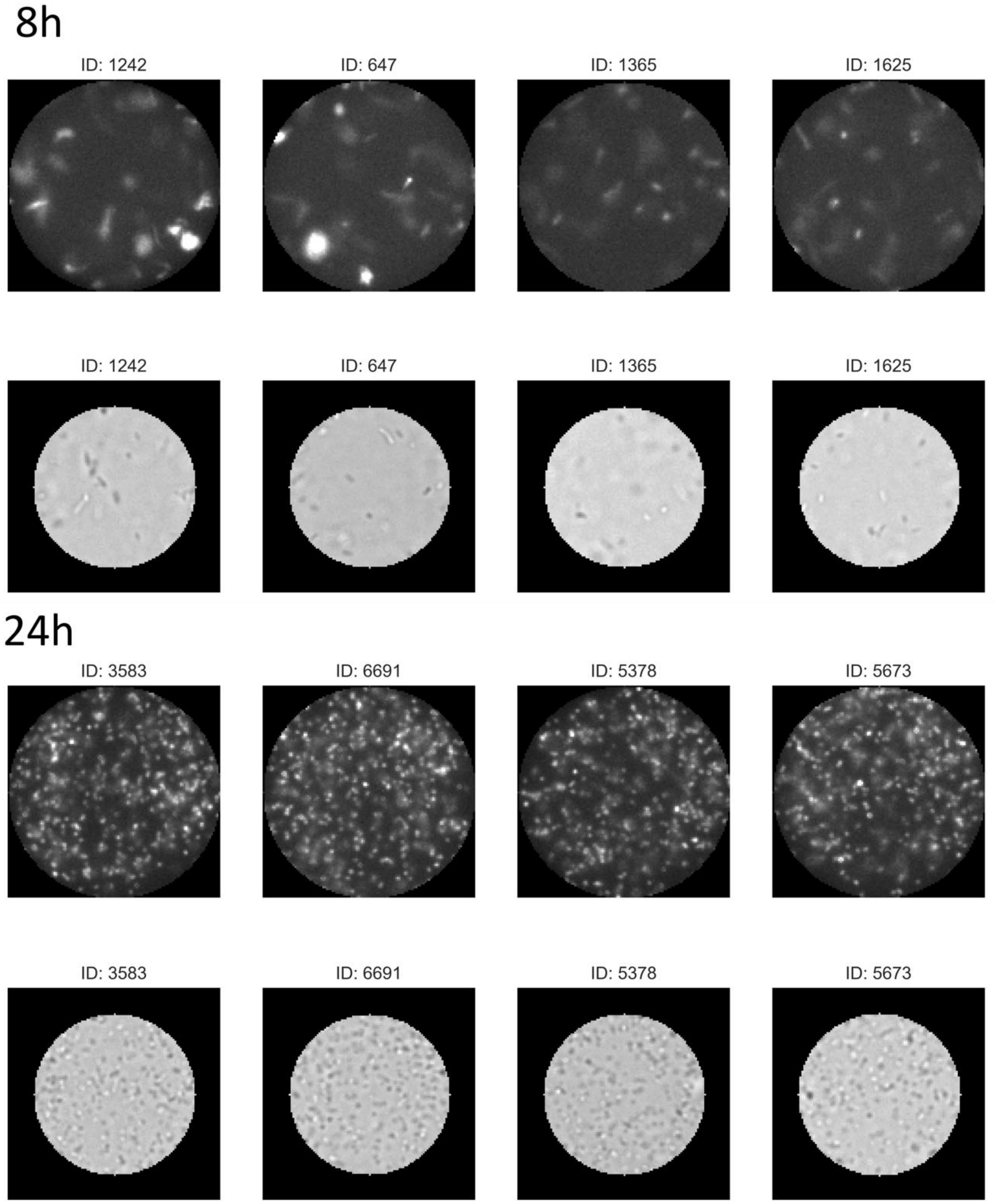

Figure S24. Example images showing selected droplets (with object counts above 10; i.e., the number of bacterial cells within every droplet) in the presence of streptomycin at 0.35 µg mL^{-1} after 8 and 24 hours of incubation in YFP (upper images) and brightfield (lower images). The images show multiple non-filamentous cells, probably released by aggregate dispersal.

Streptomycin susceptibility assays for biological replicate 2

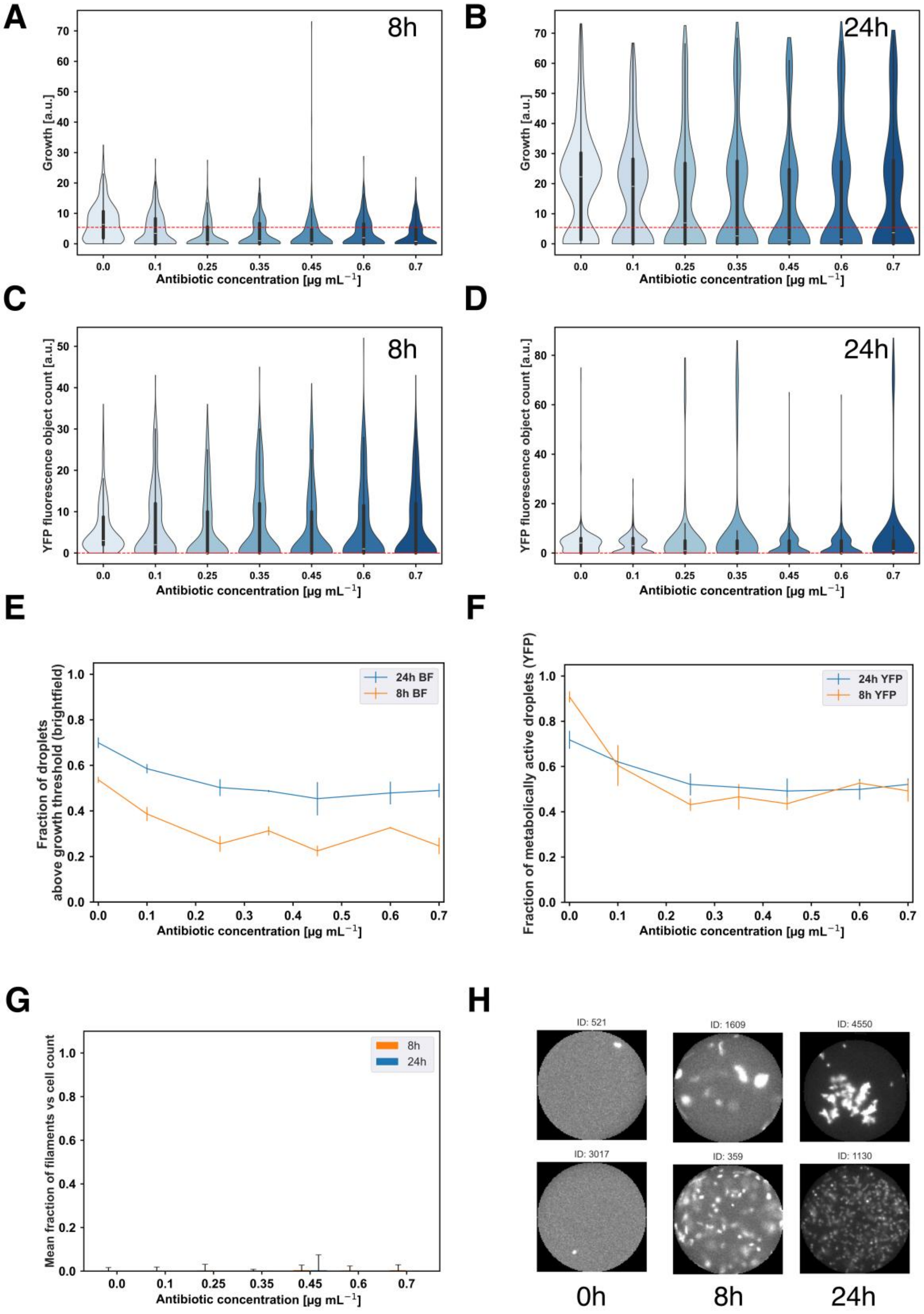

Figure S25. Streptomycin susceptibility assays for *E. coli* K12 strain RJA002, corresponding to biological replicate two. Droplet experiments were conducted in the sub-inhibitory range of

0.1 to 0.7 µg mL^{-1} streptomycin. Violin plots for the growth of droplets exposed to different streptomycin concentrations based on brightfield image analysis after 8 and 24 hours of incubation are shown in (A) and (B). A threshold line (dashed red line) is defined (=3.4 and 5.4 for biological replicates 1 and 2, respectively) to distinguish empty and filled droplets. (C) and (D) demonstrate the YFP object count distributions at the two time points, that is, the distribution of cell counts. (E) shows the fraction of droplets exhibiting growth higher than the designated brightfield growth threshold. (F) shows the fraction of metabolically active droplets, defined as those whose YFP object count is greater than zero. Error bars indicate the standard deviation, calculated by randomly splitting the dataset for each antibiotic concentration into three sub-sets. The fraction of filamentous cells within droplets, for different antibiotic concentrations, is shown in (G), and error bars indicate the standard deviation across droplets, cut at zero. (H) shows randomly picked images of streptomycin-containing droplets at 0.35 µg mL^{-1}, at different time points. Minimum droplet number n = 307 and maximum n = 585.

Droplet images of the ampicillin populations

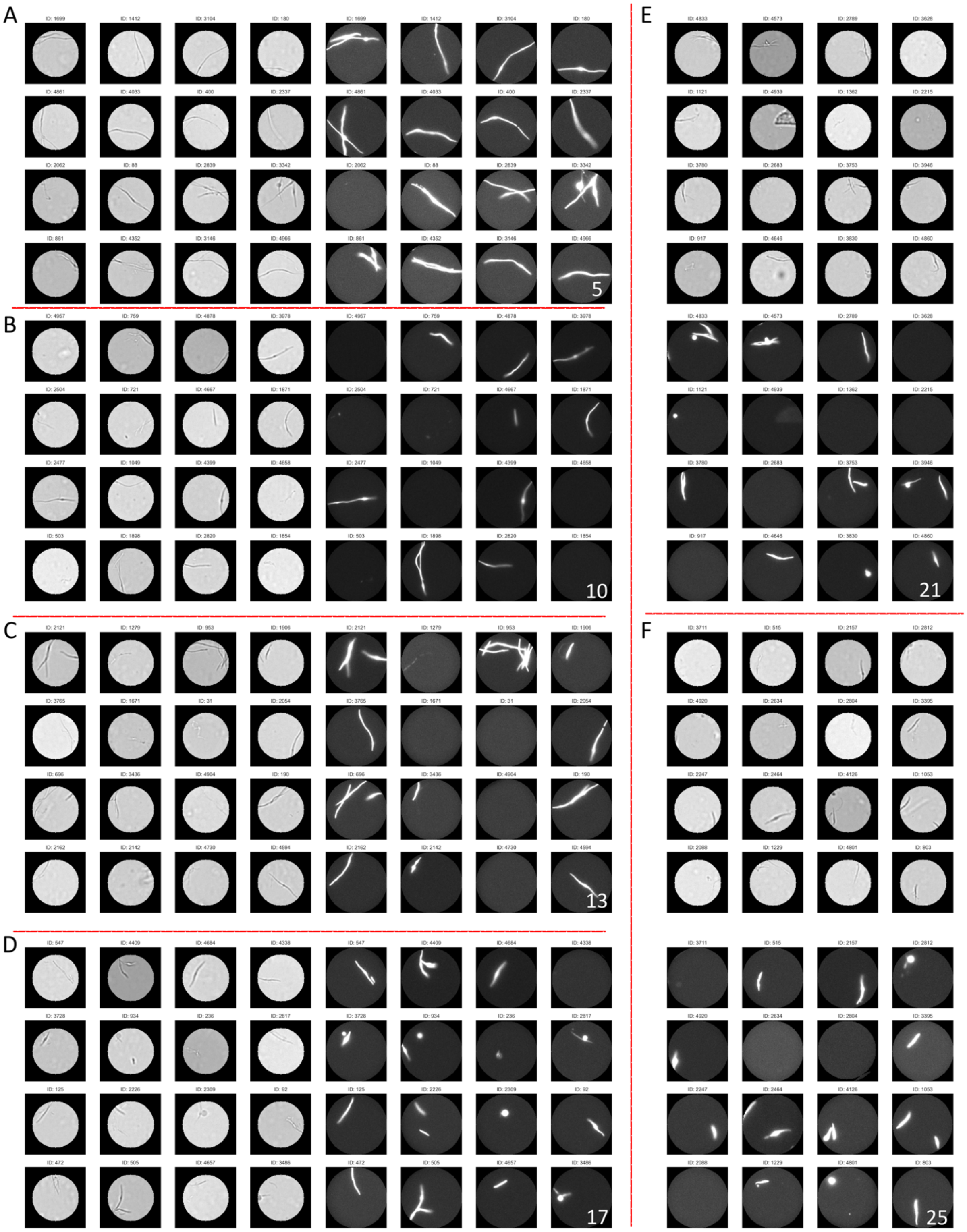

Figure S26. Droplet images corresponding to different ampicillin concentrations, after 8 hours of incubation, in the bright field and YFP channels. The droplet images are randomly chosen, but only droplets that show non-zero growth (based on bright field growth data and thresholding conditions for each replicate; please check materials and methods) are shown. The numbers in each set of images indicate the antibiotic concentration in µg mL^{-1}.

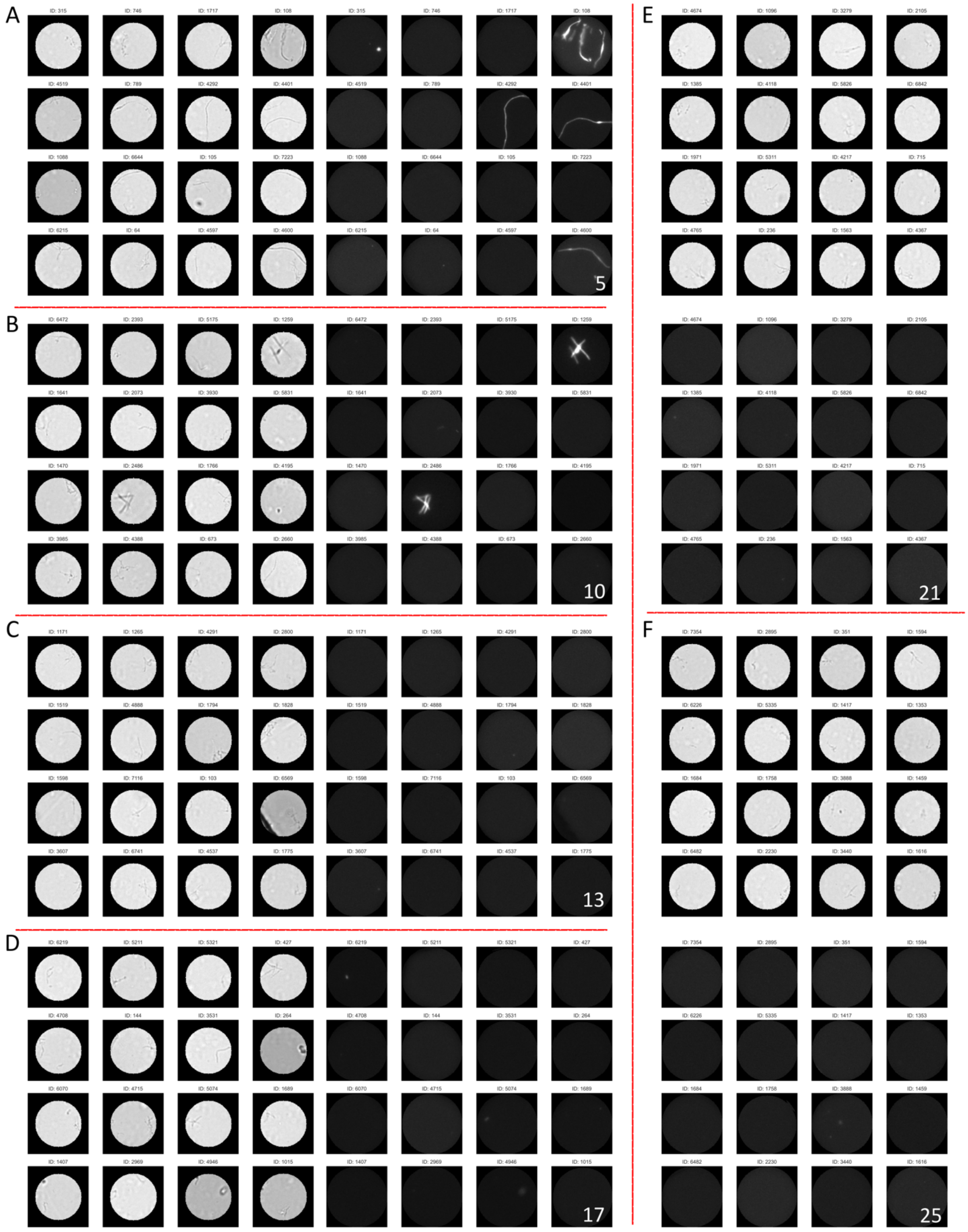

Figure S27. Droplet images corresponding to different ampicillin concentrations, after 24 hours of incubation, in the bright field and YFP channels. The droplet images are randomly chosen, but only droplets that show non-zero growth (based on bright field growth data and thresholding conditions for each replicate; please check materials and methods) are shown. The numbers in each set of images indicate the antibiotic concentration in μg mL^{-1}.

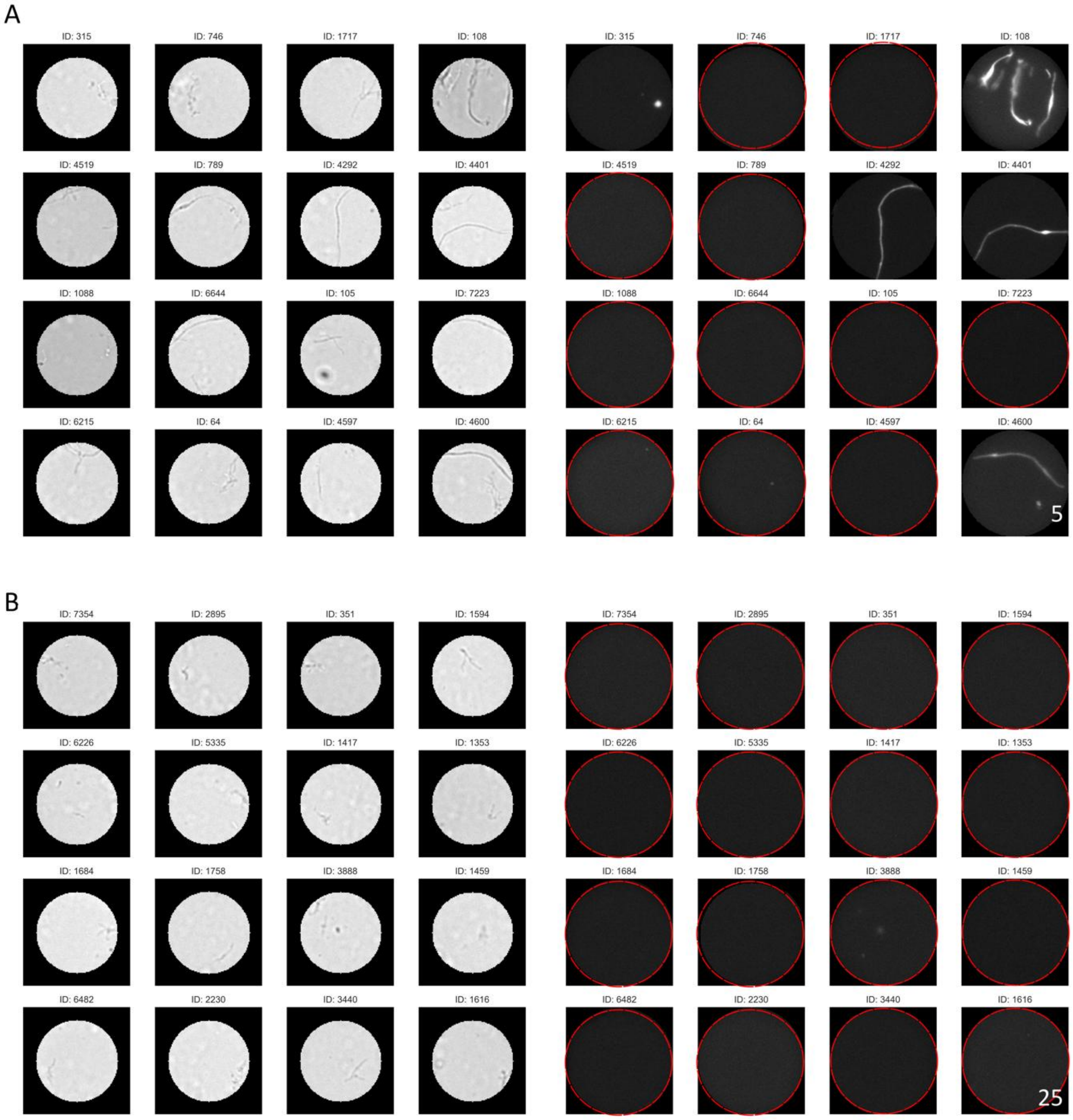

Figure S28. Images of droplets with non-zero growth (based on bright field growth data) are shown for two extreme ampicillin concentrations: 5 and 25 µg mL^{-1}, after 24 hours of incubation. Images are shown in the bright field and YFP channels. (A) shows droplets at 5 µg mL^{-1}, and (B) shows droplets at 25 µg mL^{-1}. Most bacterial cells within the droplets experience lysis, as shown by the YFP image intensities (red circles show droplet outlines). Numbers in each set of images are antibiotic concentration in µg mL^{-1} unit.

Ampicillin susceptibility assays for biological replicate 2

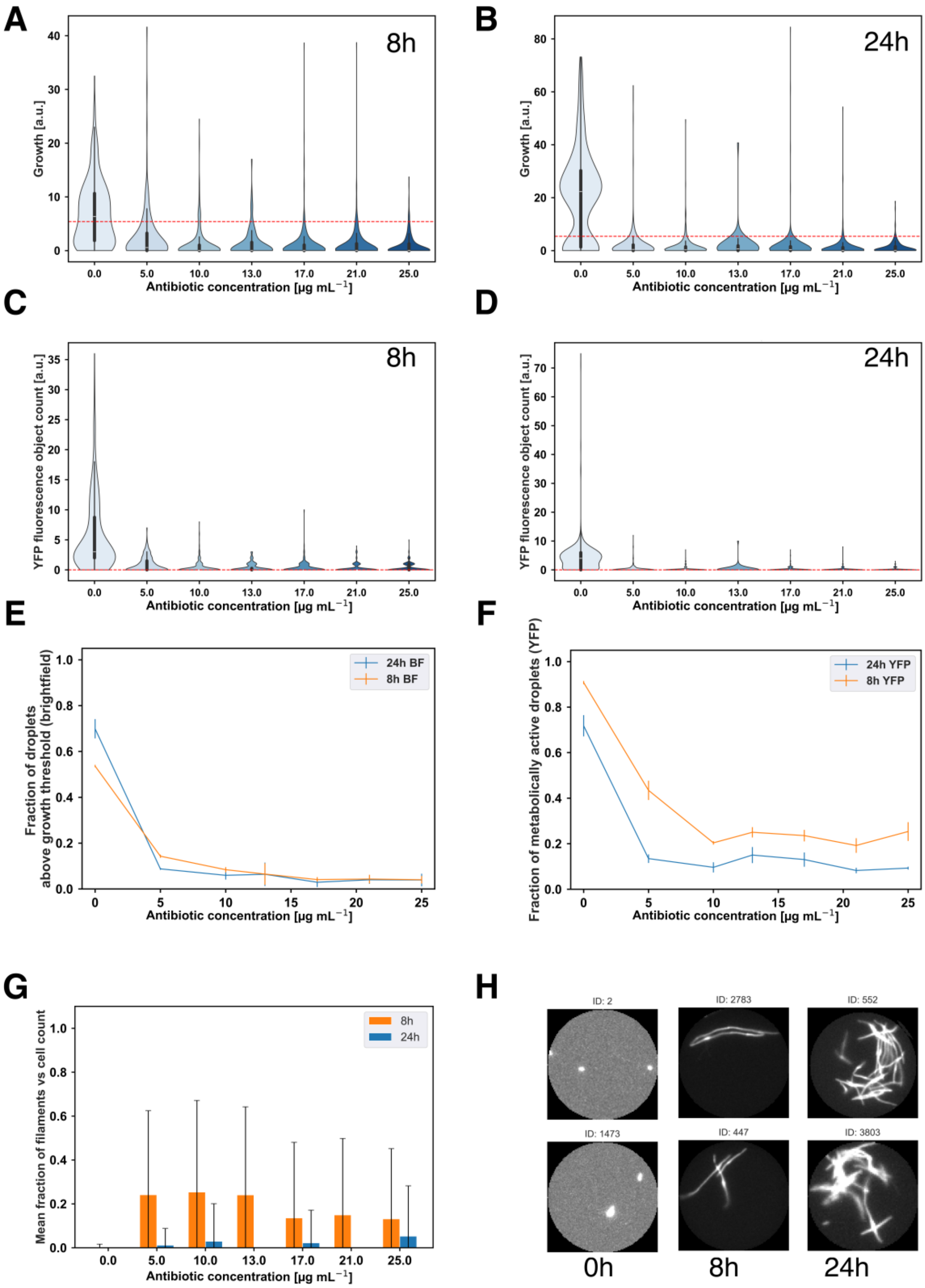

Figure S29. Ampicillin susceptibility assays for *E. coli* K12 strain RJA002 corresponding to biological replicate two. Droplet experiments were conducted in the sub-inhibitory range of 5 to 25 µg mL^{-1} ampicillin. Violin plots for the growth of droplets exposed to different ampicillin concentrations, based on brightfield analysis of images after 8 and 24 hours of incubation, are shown in (A) and (B). A threshold value is used to separate the empty and growth-exhibiting droplets in the bright field image

analysis, which is shown as a dashed red line in the violin plots. YFP cell count violin plots are shown in (C) and (D). (E) shows the fraction of droplets exhibiting growth higher than the threshold based on the brightfield images. (F) shows the fraction of metabolically active droplets, i.e. droplets with non-zero object counts based on the YFP images. Error bars indicate the standard deviation, calculated by randomly splitting the dataset for each antibiotic concentration into three sub-sets. The fraction of filamentous cells within droplets, for different antibiotic concentrations, is shown in (G), and error bars indicate the standard deviation across droplets, cut at zero. (H) shows randomly picked images of ampicillin droplets at 5 μg mL^{-1}, for different time points. Minimum droplet number n = 47 and maximum n = 585.

Brightfield growth analysis of ampicillin-treated droplets

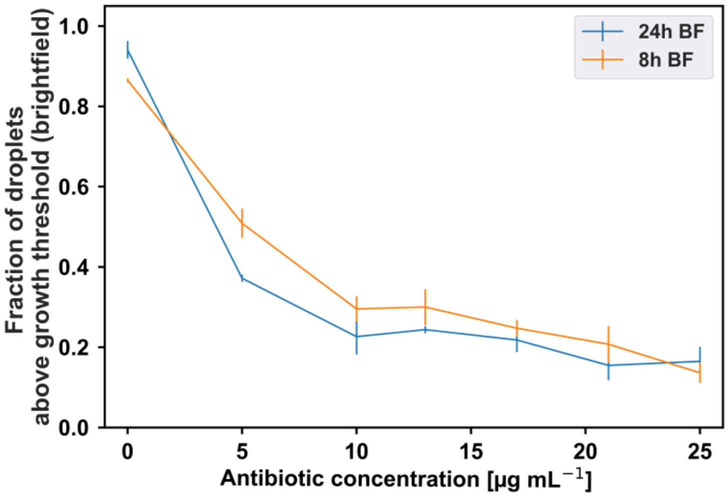

Figure S30. Fraction of droplets exhibiting growth higher than the brightfield threshold in our ampicillin experiments, for biological replicate 1. Minimum droplet number n = 177 and maximum n = 422. Error bars show the standard deviation calculated after randomly dividing the dataset of each experimental condition into three subsets.

Antibiotic leakage evaluation

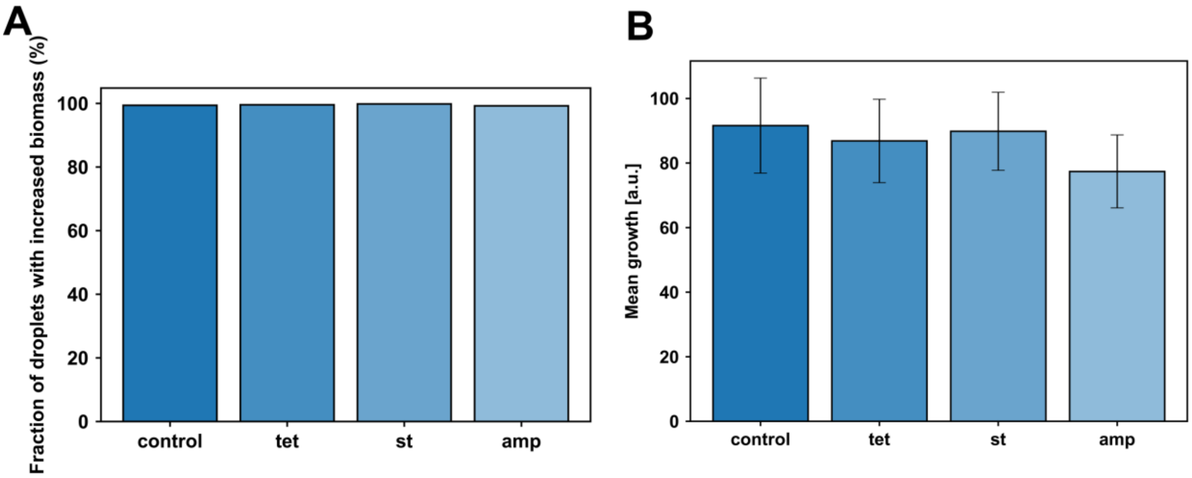

Figure S31. Antibiotic leakage evaluation. To assess whether antibiotics and the concentrations used in our investigations can have adverse effects on the model bacterial strain, we evaluated its growth

under four conditions. A control condition where all droplets are occupied only with the model bacterial strain, and three antibiotic conditions. In antibiotic conditions, droplets encapsulating the model strain are mixed with droplets containing the highest concentration of each antibiotic tested in this investigation. Using the brightfield images of the control population at the 0-hour time point, we defined a threshold to identify droplets with increased biomass after 24 hours across all experimental conditions (see the *Antibiotic Leakage Evaluation* part of the Experimental Section). Panel **A** shows a bar plot of the fraction of droplets with increased biomass for each condition. 500 data points were randomly selected from the pool of droplets, with 5450, 713, 1646, and 1943 droplets for the control, tetracycline (tet), streptomycin (st), and ampicillin (amp) conditions, respectively. The fractions are 100%, 99.8%, 100%, and 98.6% for control, tetracycline, streptomycin, and ampicillin, respectively. Panel **B** shows the mean growth value, with error bars indicating the standard deviation.
